# Global update on the susceptibilities of human influenza viruses to neuraminidase inhibitors and the cap-dependent endonuclease inhibitor baloxavir, 2018–2020

**DOI:** 10.1016/j.antiviral.2022.105281

**Published:** 2022-03-12

**Authors:** Elena A. Govorkova, Emi Takashita, Rod S. Daniels, Seiichiro Fujisaki, Lance D. Presser, Mira C. Patel, Weijuan Huang, Angie Lackenby, Ha T. Nguyen, Dmitriy Pereyaslov, Aine Rattigan, Sook Kwan Brown, Magdi Samaan, Kanta Subbarao, Sun Wong, Dayan Wang, Richard J. Webby, Hui-Ling Yen, Wenqing Zhang, Adam Meijer, Larisa V. Gubareva

**Affiliations:** a WHO Collaborating Centre for Studies on the Ecology of Influenza in Animals and Birds, St. Jude Children’s Research Hospital, Memphis, TN, 38105-3678, USA; b WHO Collaborating Centre for Reference and Research on Influenza, National Institute of Infectious Diseases, Gakuen 4-7-1, Musashimurayama, Tokyo, 208-0011, Japan; c WHO Collaborating Centre for Reference and Research on Influenza, The Francis Crick Institute, Worldwide Influenza Centre, 1 Midland Road, London, NW1 1AT, United Kingdom; d National Institute for Public Health and the Environment, PO Box 1, 3720, BA, Bilthoven, the Netherlands; e WHO Collaborating Centre for Surveillance, Epidemiology and Control of Influenza, Centres for Disease Control and Prevention, 1600 Clifton RD NE, MS H17-5, Atlanta, GA, 30329, USA; f WHO Collaborating Centre for Reference and Research on Influenza, National Institute for Viral Disease Control and Prevention, China CDC, Beijing, China; g National Infection Service, Public Health England, London, NW9 5HT, United Kingdom; h Global Influenza Programme, World Health Organization, Avenue Appia 20, 1211, Geneva, 27, Switzerland; i WHO Collaborating Centre for Reference and Research on Influenza, Peter Doherty Institute for Infection and Immunity, Melbourne, Victoria, 3000, Australia; j Public Health Laboratory Centre, 382 Nam Cheong Street, Hong Kong, China; k School of Public Health, Li Ka Shing Faculty of Medicine, The University of Hong Kong, Hong Kong, China

**Keywords:** Antiviral, Neuraminidase inhibitor, Polymerase inhibitor, Baloxavir, Reduced susceptibility, Influenza

## Abstract

Global analysis of the susceptibility of influenza viruses to neuraminidase (NA) inhibitors (NAIs) and the polymerase acidic (PA) inhibitor (PAI) baloxavir was conducted by five World Health Organization Collaborating Centres for Reference and Research on Influenza during two periods (May 2018–May 2019 and May 2019–May 2020). Combined phenotypic and NA sequence-based analysis revealed that the global frequency of viruses displaying reduced or highly reduced inhibition (RI or HRI) or potential to show RI/HRI by NAIs remained low, 0.5% (165/35045) and 0.6% (159/26010) for the 2018–2019 and 2019–2020 periods, respectively. The most common amino acid substitution was NA-H275Y (N1 numbering) conferring HRI by oseltamivir and peramivir in A(H1N1)pdm09 viruses. Combined phenotypic and PA sequence-based analysis showed that the global frequency of viruses showing reduced susceptibility to baloxavir or carrying substitutions associated with reduced susceptibility was low, 0.5% (72/15906) and 0.1% (18/15692) for the 2018–2019 and 2019–2020 periods, respectively. Most (n = 61) of these viruses had I38→T/F/M/S/L/V PA amino acid substitutions. In Japan, where baloxavir use was highest, the rate was 4.5% (41/919) in the 2018–2019 period and most of the viruses (n = 32) had PA-I38T. Zoonotic viruses isolated from humans (n = 32) in different countries did not contain substitutions in NA associated with NAI RI/HRI phenotypes. One A(H5N6) virus had a dual substitution PA-I38V + PA-E199G, which may reduce susceptibility to baloxavir. Therefore, NAIs and baloxavir remain appropriate choices for the treatment of influenza virus infections, but close monitoring of antiviral susceptibility is warranted.

## 1. Introduction

Antivirals known as neuraminidase (NA) inhibitors (NAIs) and polymerase acidic (PA) inhibitor (PAI) have been approved in many countries for controlling influenza infections. The four NAIs in current use are oral oseltamivir, inhaled zanamivir, intravenous peramivir, and inhaled laninamivir (approved only in Japan). The orally administered PAI baloxavir marboxil (baloxavir) is the first in a new class of drugs targeting the cap-dependent endonuclease activity of the influenza PA protein. This antiviral was approved in Japan and the United States in 2018 and is now approved in several countries (Beigel and Hayden, 2021; Ison et al., 2021). Antiviral usage against influenza differs among countries, with the highest usage per capita being reported in Japan (Table S1). Oseltamivir remains the most widely used influenza antiviral and is now available as a generic drug.

The emergence and spread of viruses with reduced susceptibility to antivirals can diminish the usefulness of these drugs for controlling influenza infections. This necessitates continuous monitoring of antiviral susceptibility among circulating viruses. Antiviral testing has become an integral part of influenza virologic surveillance conducted by laboratories of the World Health Organization (WHO) Global Influenza Surveillance and Response System (GISRS). Members of the WHO GISRS Antiviral Working Group (WHO-AVWG) compile and review data collected throughout the year and provide regular reports (Gubareva et al., 2017; Hurt et al., 2016; Lackenby et al., 2018; Meijer et al., 2014; Takashita et al., 2015a, 2020a).

NAI susceptibility is assessed using the MUNANA (4-(methyl-umbelliferyl)-N-acetylneuraminic acid) fluorescence-based NA enzyme inhibition assay, with minor modifications in each laboratory, to determine 50% inhibitory concentrations (IC_50_). Tokyo WHO Collaborating Centre (CC) uses a chemiluminescent-based assay (NA-XTD^™^ Influenza Neuraminidase Assay Kit, Applied Biosystems) to assess a small subset of viruses with low NA activity. As there are no established cut-offs defining drug resistance, and to harmonize phenotypic data across WHO CCs, the WHO-AVWG criteria for reporting NAI susceptibility data are based on comparison to the median IC_50_ value of the respective type/subtype/lineage (World Health Organization, 2012). These criteria differ for influenza A and B viruses: normal inhibition (NI) (<10-fold for type A viruses; <5-fold for type B viruses), reduced inhibition (RI) (10- to 100-fold for type A viruses; 5- to 50-fold for type B viruses), and highly reduced inhibition (HRI) (>100-fold for type A viruses; >50-fold for type B viruses). RI or HRI phenotypes of influenza viruses are associated with amino acid substitutions or deletions at conserved residues that form/stabilize the active site of the NA (Lee and Hurt, 2018). Overall, the frequency of influenza viruses with RI/HRI in global circulation has been low (<2%). However, during 2007–2009, oseltamivir resistance rose drastically among A(H1N1) viruses, being conferred by the NA-H275Y amino acid substitution (Dharan et al., 2009; Hurt et al., 2009; Meijer et al., 2009). A(H1N1)pdm09 viruses displaced the oseltamivir-resistant A(H1N1) viruses. Since then, oseltamivir-resistant A(H1N1)pdm09 viruses containing NA-H275Y have been detected sporadically, sometimes as clusters of cases but with limited circulation in communities (Hurt et al., 2012; Takashita et al., 2015b). Besides NA-H275Y, numerous other substitutions have been associated with RI/HRI phenotypes, and these have been summarized by the WHO-AVWG (NA marker table, https://cdn.who.int/media/docs/default-source/influenza/nai-reduced-susceptibility-marker-table-who_table-1.pdf).

With the advances in next-generation sequencing (NGS) technologies, there is increased emphasis on establishing sequence-based virologic surveillance, which can provide a framework for selecting viruses for phenotypic testing. Several years ago, the Atlanta WHO CC implemented the sequence-first initiative, whereby all submitted viruses are subjected to full-genome sequence analysis so that the generated sequences can be screened for various changes, including potential markers of reduced susceptibility to drugs. Other GISRS laboratories are increasing their sequencing capabilities and are sharing their findings via the Global Initiative on Sharing All Influenza Data (GISAID), thereby bolstering surveillance data generation.

Information on molecular changes that affect baloxavir susceptibility is limited. Reduced susceptibility to baloxavir is most often associated with amino acid substitution at residue 38 in the PA protein, with PA-I38T being particularly common (Omoto et al., 2018; Imai et al., 2020), and frequencies of baloxavir treatment–emergent variants differing between virus types and subtypes (Hayden et al., 2018; Ince et al., 2020; Ison et al., 2020; Uehara et al., 2020). PA substitutions associated with reduced susceptibility (PA marker) have been summarized by the WHO-AVWG (see, https://cdn.who.int/media/docs/default-source/influenza/summary-of-polymerase-acidic-(pa)-protein-amino-acid-substitutions-analysed-for-their-effects-on-baloxavir-susceptibility.pdf).

Phenotypic testing enables the detection of viruses with reduced susceptibility to drugs and combined with gene sequencing, allows identification of known and new markers. Cell culture–based assays are used to determine the baloxavir susceptibility of influenza viruses by assessing virus replication in the presence of the drug. Two assays, the high-content imaging neutralization test (HINT) and the focus reduction assay (FRA), are currently used by the Atlanta and Tokyo WHO CCs, respectively (Gubareva et al., 2019; Takashita et al., 2018). These two assays yield different effective concentrations (EC_50_) values for a given virus, but the fold changes are similar (Takashita et al., 2020a). Currently, there are no criteria for defining resistance or reduced susceptibility to baloxavir. An arbitrary threshold (cut-off) of a ≥3-fold increase in the median EC_50_ is used for reporting viruses with reduced susceptibility to baloxavir (Gubareva et al., 2019; Takashita et al., 2020a). This cut-off should capture >95% of viruses with reduced susceptibility to baloxavir (Ince et al., 2020).

National Influenza Centres (NICs) receive influenza virus–positive clinical specimens collected in their respective countries and perform initial analyses (https://www.who.int/teams/global-influenza-programme/laboratory-network/virological-surveillance). Representative numbers of influenza-positive clinical specimens and/or viruses of each genetic type/subtype/lineage are then shipped to a GISRS WHO CC for further characterization. The WHO CCs propagate viruses in MDCK, MDCK-SIAT1, or hCK cells before drug susceptibility testing (Hurt et al., 2012; Takada et al., 2019).

This is the seventh WHO-AVWG review of antiviral susceptibility, and it primarily comprises data generated by WHO CCs. It includes influenza antiviral susceptibility data for two consecutive periods, May (week 21) to May (week 20) of the following year as used in previous global reports (thereby covering Southern and subsequent Northern Hemisphere influenza seasons) for 2018–2019 and 2019–2020, with an emphasis on sequence-based analysis. Antiviral susceptibility results for NAIs and baloxavir are analyzed, keeping in mind the changing algorithms for virus testing employed by the WHO CCs. For the first time, the report includes antiviral susceptibility analysis of zoonotic viruses detected in different countries and reported to the WHO. This analysis provides data for evaluating the risk posed by viruses with pandemic potential. The emergence of the COVID-19 pandemic delayed analyses of the data presented in this report.

## 2. Neuraminidase inhibitors (NAIs)

### 2.1. Analysis of phenotypic and genotypic NAI susceptibility data from WHO CCs

**Fig. 1.**
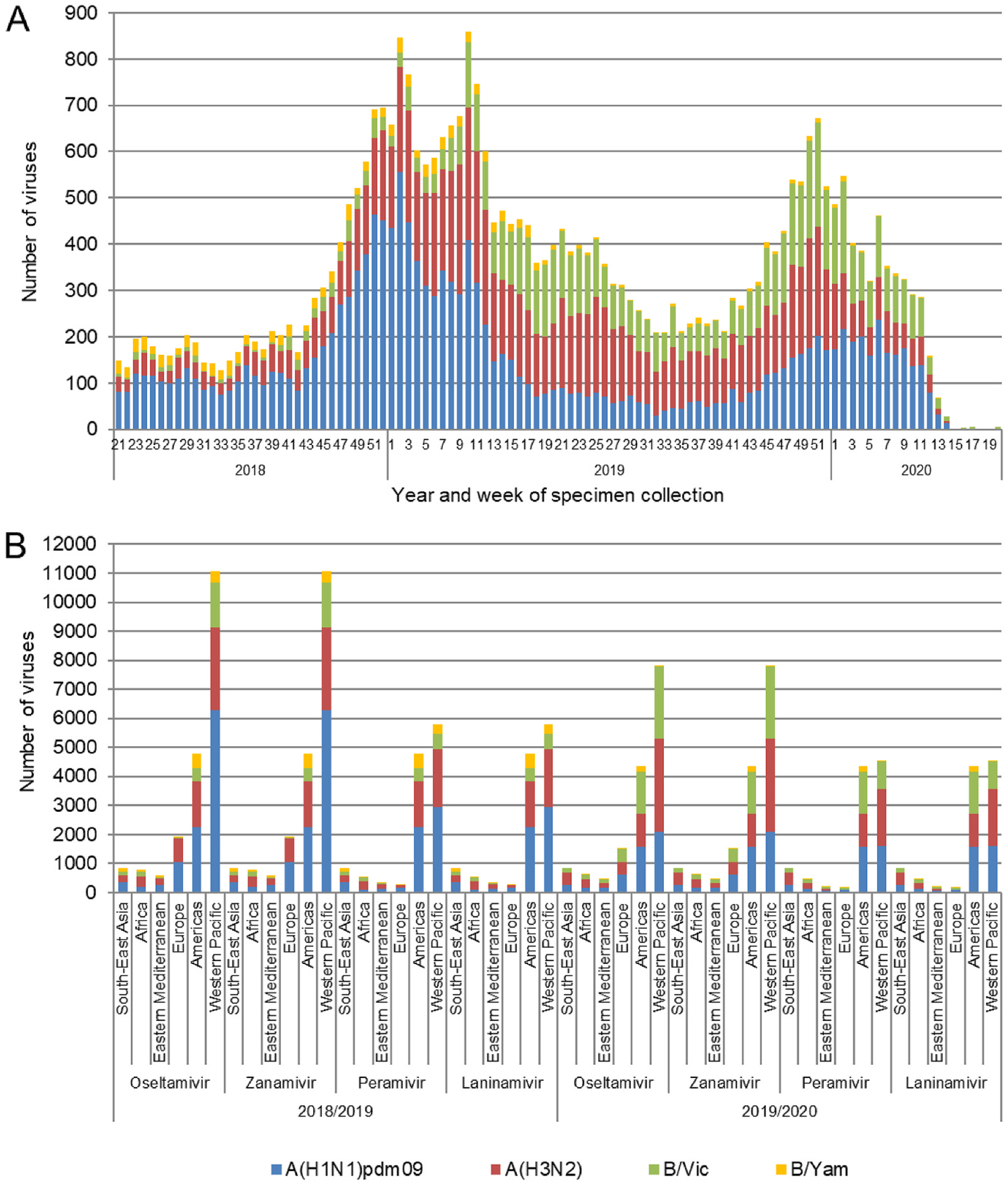
Influenza viruses collected and assessed for NAI susceptibility during 2018–2019 and 2019–2020 periods. For each period viruses were collected between week 21 and week 20 of the following year. (A) Week of specimen collection and virus type/subtype/lineage for specimens assessed in the 2018–2019 and 2019–2020 periods. Typically, week 21 to week 39 of a year covers the Southern Hemisphere influenza season, while week 40 of a year to week 20 of the following year covers the Northern Hemisphere influenza season. (B) The number of viruses assessed for susceptibility to the four NAIs using NA inhibition assays and/or sequence-based analysis, by WHO Region for the 2018–2019 and the 2019–2020 periods.

During 2018–2019 and 2019–2020 periods, a total of 19966 and 15582 viruses, respectively were assessed for NAI susceptibility by five WHO CCs using phenotypic and/or NA sequence-based methods (Figs. S1 and S2). Of these respective total numbers, 13536 in 2018–2019 and 9853 in 2019–2020 were tested phenotypically by an NA inhibition assays. The potential NAI susceptibility of remaining viruses 6430 in 2018–2019, 5729 in 2019–2020, with almost equal proportions of viruses collected in the United States and other countries were assessed by Atlanta CC based on NA sequence analysis to ensure that all viruses with previously reported markers were tested phenotypically (Fig. 1A and Table S2). All these viruses (19966 and 15582, respectively for both periods) were assessed for susceptibility to oseltamivir and zanamivir (Fig. 1B). Three WHO CCs (Atlanta, Melbourne, and Tokyo) also assessed viruses for susceptibility to peramivir and laninamivir using phenotypic and/or NA sequence-based methods. Most viruses originated from the WHO regions of the Western Pacific (WPRO; 55.4% for 2018–2019 and 50.1% for 2019–2020) and the Americas (PAHO; 23.9% for 2018–2019 and 28% for 2019–2020) (Fig. 1B). Only 20.7% of viruses for 2018–2019 and 21.9% for 2019–2020, were from the African (AFRO), Eastern Mediterranean (EMRO), European (EURO), or Southeast Asian regions (SEARO) (Fig. 1B). Based on the WHO GISRS global web-based tool for influenza virologic surveillance (https://www.who.int/initiatives/global-influenza-surveillance-and-response-system), totals of 685332 influenza viruses were detected globally and reported to FluNet for 2018–2019 and 694536 for 2019–2020, respectively. Therefore, the viruses analyzed for NAI susceptibility by the WHO CCs in this study represent 2.9% of global influenza detections reported to FluNet for 2018–2019 and 2.2% of those reported for 2019–2020.

**Fig. 2.**
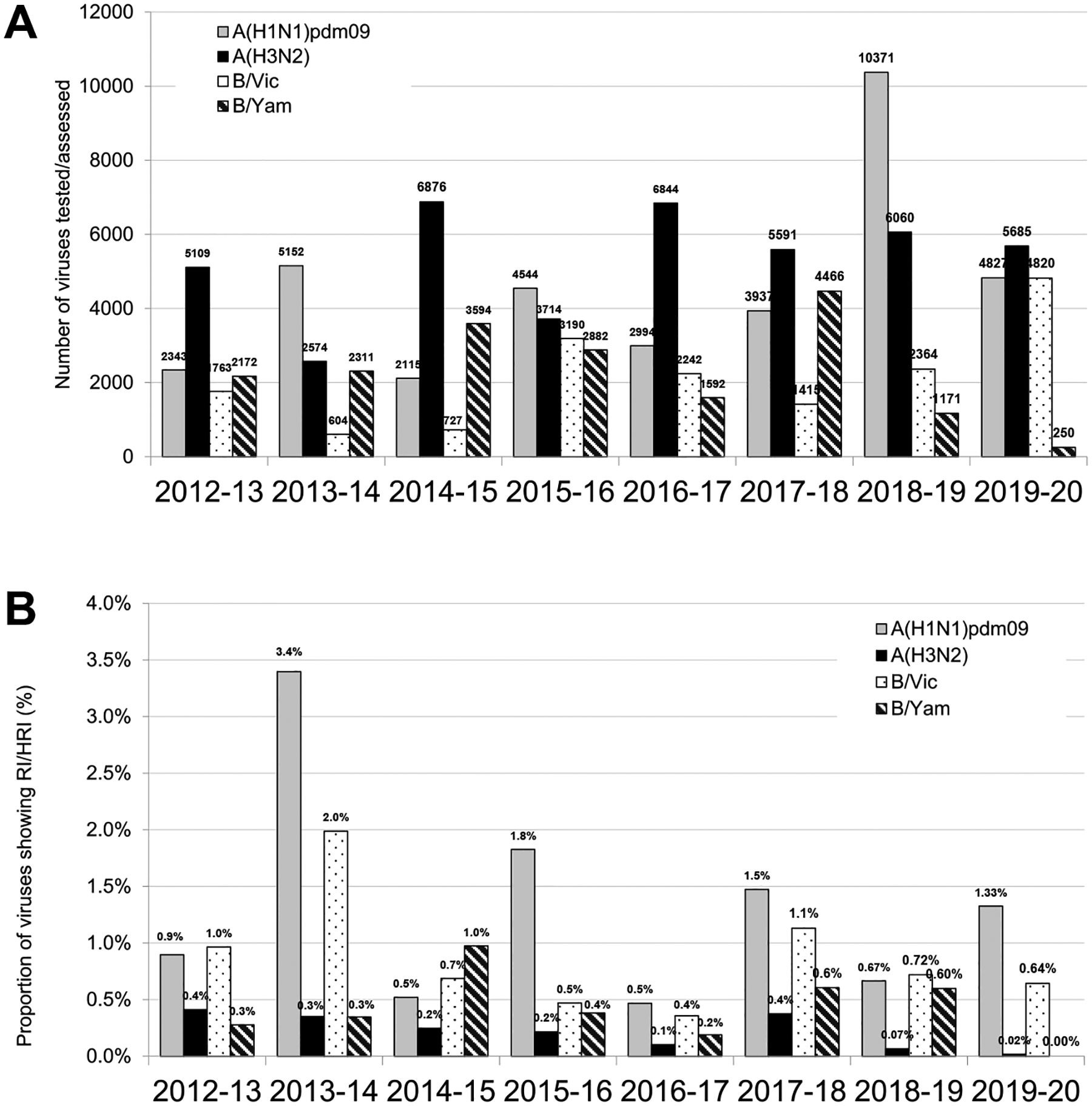
Comparison of NAI susceptibility surveillance over eight periods. (A) Number of viruses tested. For the 2012–2018 period testing was reported based on NA inhibition assays only. For the 2018–2020 period results of assessment by NA inhibition assays and/or sequence-based analysis were included. (B) The proportion of viruses showing RI/HRI by NAIs over the 2012–2020 period. Data were compiled from the global studies of viruses isolated during the 2012–2013 (Meijer et al., 2014), 2013–2014 (Takashita et al., 2015a, 2015b), 2014–2015 (Hurt et al., 2016), 2015–2016 (Gubareva et al., 2017), 2016–2017 (Lackenby et al., 2018), 2017–2018 (Takashita et al., 2020a), and 2018–2020 (current study) periods.

For 2018–2019, influenza A(H1N1)pdm09 viruses were the most prevalent among the viruses assessed (10371; 51.9%), followed by A (H3N2) (6060; 30.4%), B/Victoria-lineage (2364; 11.8%), and B/Yamagata-lineage (1171; 5.9%) viruses (Fig. 2A). For 2019–2020, the numbers of A(H1N1)pdm09 (4827; 31.0%), A(H3N2) (5685; 36.5%) and B/Victoria-lineage (4820; 30.9%) viruses assessed were similar, but B/Yamagata-lineage viruses were notably less prevalent (250; 1.6%) (Fig. 2A).

Overall, phenotypic testing identified 97/13536 viruses [7392 A (H1N1)pdm09, 3884 A(H3N2), 1672 B/Victoria-lineage, and 588 B/Yamagata-lineage] viruses from 2018–2019 and 96/9853 [2888 A (H1N1)pdm09, 3871 A(H3N2), 3029 B/Victoria-lineage, and 65 B/Yamagata-lineage] from 2019–2020 with RI/HRI. Sequence analysis of additional viruses, 6430 [2979 A(H1N1)pdm09, 2176 A(H3N2), 692 B/Victoria-lineage, and 583 B/Yamagata-lineage] for 2018–2019 and 5729 [1939 A(H1N1)pdm09, 1814 A(H3N2), 1791 B/Victoria-lineage, and 185 B/Yamagata-lineage] for 2019–2020 identified no NA substitutions associated with RI/HRI phenotypes. Thus, 97/19966 (0.5%) viruses assessed for 2018–2019 and 96/15582 (0.6%) assessed for 2019–2020 were identified as having RI/HRI by at least one NAI; minor decreases compared to the corresponding result (0.8%) for 2017–2018 (Takashita et al., 2020a).

### 2.2. A(H1N1)pdm09 viruses showing RI/HRI

**Fig. 3.**
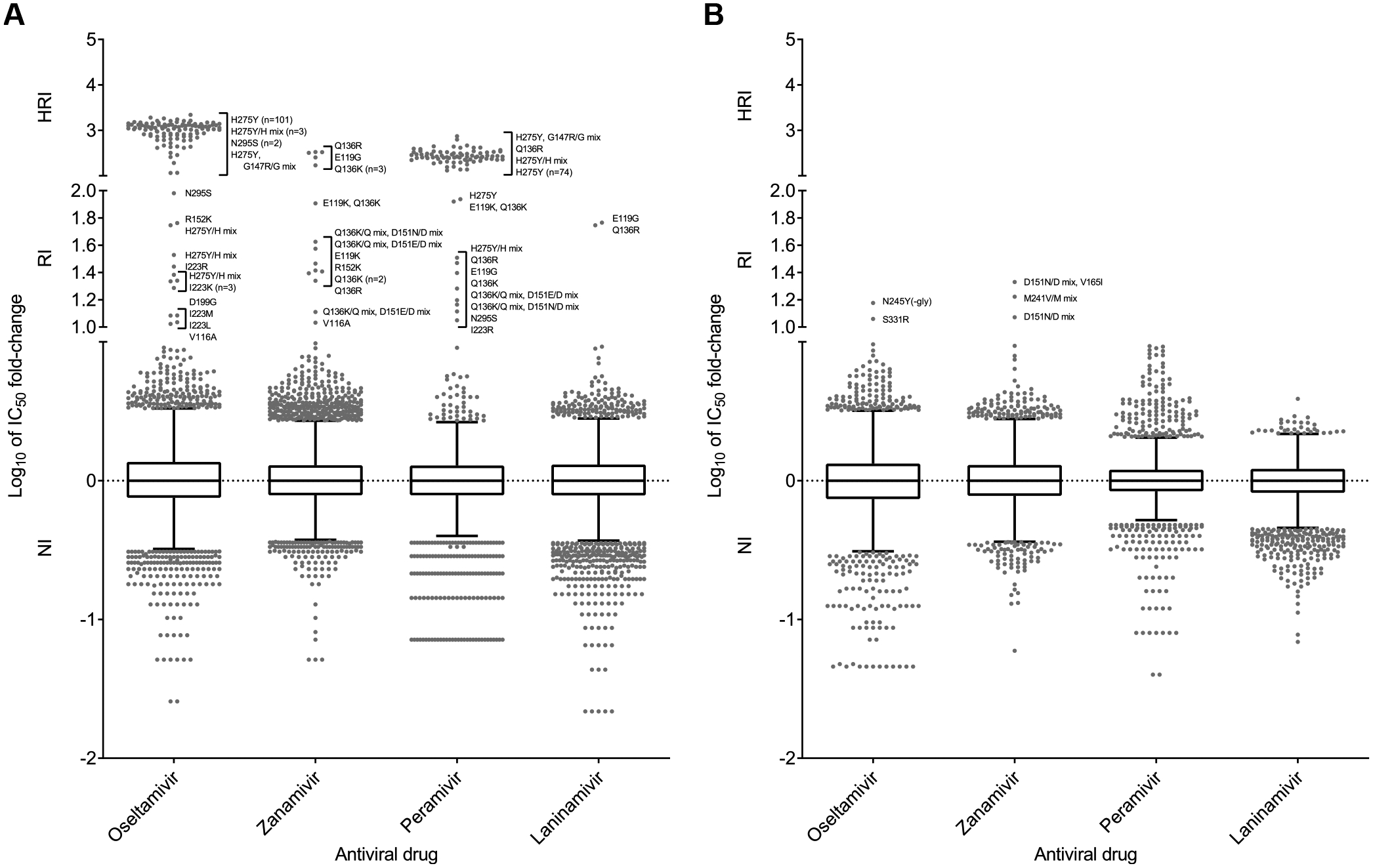

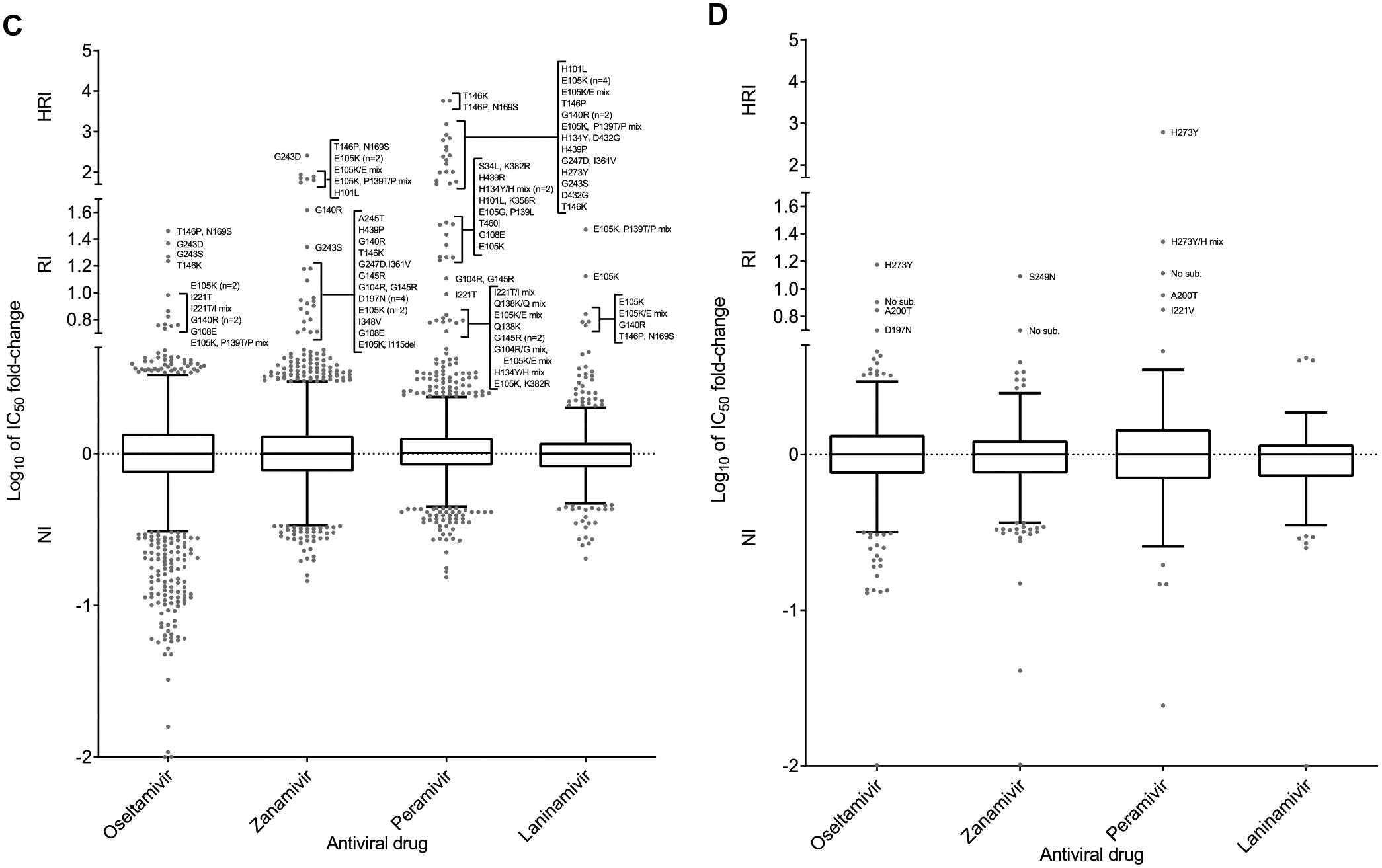
Column-scatter plots of log-transformed 50% inhibitory concentration (IC_50_) fold-change values for NAIs. Overall, 13536/19966 and 9853/15582 viruses were tested phenotypically for 2018–2019 and 2019–2020, respectively. Data are presented by virus subtype or lineage [(A) A(H1N1)pdm09; (B) A(H3N2); (C) B/Victoria-lineage; and (D) B/Yamagata-lineage] and NAI (labelled on the x-axis: oseltamivir, zanamivir, peramivir and laninamivir). The boxes indicate the 25th–75th percentiles, and the whiskers stretch to the lowest and highest values within 1.5 times the interquartile region (IQR) value from both the 25th and 75th percentile values, respectively (Tukey’s definition). The y-axes have been split into three compartments according to the thresholds recommended by the World Health Organization Expert Working Group of GISRS for normal inhibition (NI) (<10-fold for type A viruses; <5-fold for type B viruses), reduced inhibition (RI) (10- to 100-fold for type A viruses; 5- to 50-fold for type B viruses), and highly reduced inhibition (HRI) (>100-fold for type A viruses; >50-fold for type B viruses). NA amino acid substitutions are shown for viruses displaying RI or HRI phenotypes that have been sequenced. Viruses showing NI but carrying amino acid substitutions previously associated with RI or HRI by one or more NAI or showing an RI or HRI phenotype for another NAI are indicated in grey in the NI area above 1.5 times the IQR from the 75th percentile border and below the RI threshold value. Full details about these viruses are given in Tables S3 and S4. Amino acid position numbering is specific to A subtype and B type. Most viruses were tested for susceptibility to oseltamivir and zanamivir; only a subset was tested for susceptibility to peramivir and laninamivir.

In 2018–2019 and 2019–2020, 69/10371 (0.7%) and 64/4827 (1.3%) viruses, respectively, exhibited RI/HRI by at least one NAI (Fig. 2A and B), indicating an increase in the incidence of RI/HRI viruses in 2019–2020. Most viruses with a RI/HRI phenotype contained the NA-H275Y substitution (n = 101, 76%) and showed the expected increases in IC_50_ for oseltamivir and peramivir (Table 1 and Fig. 3A). In addition, six viruses with a NA-H275Y/H mixture showed elevated IC_50_ for oseltamivir and peramivir (Table 1). These NA-H275Y variants were collected in 15 countries (Table S3). For 71/101 viruses, NA-H275Y was confirmed in the corresponding clinical specimens; no clinical specimens were available for the remaining 30 viruses (Tables 1 and S3). Of the 85 patients with available clinical history, 42 were outpatients and 43 were hospitalized (Table 1). Antiviral treatment history was available for 60/101 patients, including 29 patients who did not receive an NAI before specimen collection (Tables 1 and S3). Immunocompromised status was reported for five patients who shed NA-H275Y viruses (Table 1).

Three viruses with NA-I223K substitution showed RI by oseltamivir, and the substitution was confirmed in the two corresponding clinical specimens available (Tables 1 and S3). A new substitution at this residue, NA-I223L, conferring a similar effect was also found in one clinical specimen/virus pairing. Two viruses recovered from patients with unknown treatment histories had either NA-I223M or NA-I223R substitution and exhibited RI by oseltamivir and/or peramivir. One virus from India had the dual substitution NA-H275Y + NA-G147R/G and displayed a 1376-fold increase in the IC_50_ for oseltamivir and a 746-fold increase in the IC_50_ for peramivir (Tables 1 and S3). No treatment history was available for this case, but the same dual substitution was previously reported in connection with NAI treatment (Takashita et al., 2016). Three viruses had NA-N295S and exhibited RI/HRI by oseltamivir, with the corresponding change being confirmed in the two clinical specimens available.

One virus displayed RI by oseltamivir and zanamivir, which was conferred by the substitution NA-V116A (clinical specimen not available). This substitution was previously reported in A(H5N1) virus (Boltz et al., 2010; Hurt et al., 2007). A virus with NA-R152K showed RI by oseltamivir and zanamivir; the antiviral treatment history of this patient was unknown.

It is worth noting that several NA substitutions that confer RI/HRI, especially by zanamivir, have been linked to virus culture (NA marker table; Little et al., 2015). Among these viruses were those isolates containing NA-Q136R or NA-Q136K substitution or NA-Q136K/Q + NA-D151E/D or D151N/D mixtures (Table 1). In addition, an isolate with NA-D199G substitution displayed RI by oseltamivir, two viruses containing NA-E119K substitution (in one case combined with NA-Q136K) displayed RI by zanamivir, and one virus with NA-E119G substitution exhibited HRI by zanamivir and RI by peramivir and laninamivir (Table 1). These substitutions were not found in the matching clinical specimens.

### 2.3. A(H3N2) viruses showing RI/HRI

As in previous periods, the frequency of A(H3N2) viruses showing RI/HRI by any NAI remained very low (Fig. 2B and Table 1). 4/6060 (0.07%) viruses assessed for 2018–2019 and 1/5685 (0.02%) for 2019–2020 exhibited RI by at least one NAI (Tables 1 and S3).

One virus isolated from a hospitalized peramivir-treated patient displayed RI by oseltamivir and had NA-N245Y substitution in both the isolate and the matching clinical specimen; this substitution was not reported previously (Fig. 3B). Another virus showing RI by oseltamivir was isolated from an immunocompetent patient who had received no NAI treatment; NA-S331R substitution was found in both the isolate and the clinical specimen. A(H3N2) viruses with this change were reported previously and were characterized by a borderline NI/RI phenotype (Takashita et al., 2020a). Indeed, it has been shown that A(H3N2) viruses carrying positively-charged amino acid substitutions at NA positions 329, 331 or 334 can confer an apparent RI phenotype, but this is caused by markedly higher K_m_s for MUNANA and K_i_ values for NAIs (Hussain et al., 2021). It is possible that antibody pressure is responsible for selection of amino acid substitutions at these three positions (Air et al., 1985). Three viruses exhibiting RI by zanamivir had either NA-D151N/D mixture (in one case combined with NA-V165I) or NA-M241V/M mixtures (Tables 1 and S3). These changes are likely to have arisen during virus culture.

### 2.4. B/Victoria-lineage viruses showing RI/HRI

For B/Victoria-lineage viruses, 17/2364 (0.7%) viruses assessed for 2018–2019 and 31/4820 (0.6%) for 2019–2020 exhibited RI/HRI by at least one NAI (Fig. 2B); minor decreases compared to the corresponding result (1.1%) for 2017–2018.

These RI/HRI variants were isolated from 14 countries (Table S4), and most displayed RI/HRI by peramivir (Fig. 3C). A new NA-G145R substitution that conferred a borderline NI/RI phenotype for zanamivir was found in two viruses and in one of the two corresponding clinical specimens (Tables 2 and S4). The substitution NA-G243S was identified in a clinical specimen and its respective isolate, conferring RI/HRI by all NAIs except laninamivir. A different substitution at this residue, NA-G243D, conferred RI/HRI by oseltamivir and zanamivir; data not available for the other two NAIs. A virus with dual substitutions, NA-G247D + NA-I361V, displayed RI by zanamivir and HRI by peramivir; both substitutions were present in the clinical specimen. Based on its location, NA-G247D alone could reduce inhibition by NAIs. Numerous viruses collected in different parts of the world had previously reported substitutions (NA-D197N, NA-I221T, NA-H273Y and NA-D432G) and displayed expected changes in inhibition by NAIs.

The substitution NA-E105K or NA-E105K/E mixture was found in 11 viruses, alone or combined with other changes (i.e., NA-G104R/G mixture, NA-I115 deletion, NA-P139T/P mixture, or K382R substitution), with some viruses displaying RI/HRI by NAIs. Another substitution at this residue, NA-E105G, combined with NA-P139L substitution, conferred RI by peramivir. Notably, the majority of corresponding matching clinical specimens did not have either NA-E105K or NA-E105G. Similarly, substitutions at neighboring residues (i.e., NA-H101L and NA-G108E) that conferred RI/HRI to peramivir were not confirmed in clinical specimens or they were not available. The following substitutions conferred RI/HRI but were not found in the clinical specimens: NA-T146K or P, NA-Q138K, NA-A245T, NA-T460I). It appears that certain cell culture and virus propagation conditions favor the rapid selection of type B virus NA variants, which present challenges for phenotypic testing.

### 2.5. B/Yamagata-lineage viruses showing RI/HRI

For B/Yamagata-lineage viruses, 7/1171 (0.6%) viruses assessed for 2018–2019 and 0/250 (0%) for 2019–2020 exhibited RI/HRI by at least one NAI (Fig. 2A). These seven RI/HRI variants were collected in five countries (Table S4). A virus from Japan with an NA-H273Y substitution showed RI by oseltamivir and HRI by peramivir (Takashita et al., 2020b), and another virus from the United States with a NA-H273Y/H mixture showed RI by peramivir only (Tables 2 and S4). NA-H273Y was detected in the corresponding clinical specimens. Two viruses, one with NA-A200T substitution and one with NA-I221V substitution, displayed RI by peramivir (Fig. 3D). A virus with NA-S249N substitution (not previously reported) showed RI by zanamivir, but no clinical specimen was available. A virus with an NA-D197N substitution showed borderline NI/RI by oseltamivir.

### 2.6. Frequency of NA amino acid substitutions associated with RI/HRI by NAIs in sequence databases

We analyzed NA sequences of viruses collected during the 2018–2019 (23649 viruses) and the 2019–2020 periods (21706 viruses) that were deposited in the GISAID. According to strain designation, 8570 sequences from 2018–2019 and 11278 from 2019–2020 belonged to viruses submitted by five WHO CCs for NAI susceptibility analysis. For the remaining 15079 viruses [6765 A(H1N1)pdm09, 7033 A(H3N2), 893 B/Victoria-lineage and 388 B/Yamagata-lineage] from 2018–2019 and 10428 viruses [4195 A(H1N1)pdm09, 2480 A(H3N2), 3606 B/Victoria and 147 B/Yamagata] from 2019–2020, NA sequences were analyzed for the presence of substitutions previously associated with RI/HRI. For 2018–2019, 68 viruses [0.5% of the total; 54 A(H1N1)pdm09, six A(H3N2), six B/Victoria-lineage, and two B/Yamagata-lineage] and, for 2019–2020, 63 viruses [0.6% of the total; 52 A(H1N1)pdm09, four A (H3N2) and seven B/Victoria-lineage] were identified with NA substitutions associated with RI/HRI (Table S5).

Of the 10960 A(H1N1)pdm09 sequences analyzed for both seasons, 89 (0.8%) contained NA-H275Y (nine had an NA-H275Y/H mixture and one had additional D199N/D polymorphism). Notably, an increased frequency of A(H1N1)pdm09 viruses with potential RI/HRI in 2019–2020 (1.2%, 52/4195) was observed, compared to 2018–2019 (0.8%, 54/6765), based on analysis of sequences available in GISAID which matches frequencies of A(H1N1)pdm09 viruses analyzed by WHO CCs displaying RI/HRI (1.3% for 2019–2020 vs 0.7% for 2018–2019) (section 2.2.). Among these, three NA-H275Y viruses belonged to a cluster of oseltamivir-resistant viruses detected in the United States (Mohan et al., 2021). Eight NA sequences had substitutions at residue 223 (NA-I223M/K/R/T), and three had NA-S247G substitutions. Four other sequences showed substitutions at residues 152 (NA-R152K) or 199 (NA-D199E) or 295 (NA-N295S), and two sequences had cell culture–derived substitutions at residue 136.

Of the 9513 A(H3N2) sequences analyzed for both seasons, 10 (0.1%) contained NA substitutions associated with RI/HRI: seven had substitutions at residue 119 (NA-E119V/G), one had NA-R292K substitution, and two others had substitutions at residues 249 (NA-K249E) or 391 (NA-Q391K).

Of the 4499 B/Victoria-lineage sequences analyzed for both seasons, 13 (0.3%) had NA substitutions associated with RI/HRI: five had NA-K360E, four had NA-D197N, two had NA-I221T and two had either NA-Y142H or NA-G145E. For both seasons, a total of 535 NA sequences were analyzed for B/Yamagata-lineage viruses. Only two (0.4%) of the sequences from 2018–2019 had either NA-G145E or NA-S246P substitution associated with RI/HRI.

Overall, a combined analysis using phenotypic and/or sequence-based methods revealed that the global frequency of influenza viruses either displaying RI/HRI or with potential to exhibit RI/HRI by NAIs was low, being 0.5% (165/35045) and 0.6% (159/26010) for 2018–2019 and 2019–2020, respectively.

## 3. Cap-dependent endonuclease inhibitor

### 3.1. Combined analysis of genotypic and phenotypic baloxavir susceptibility data

During the periods covered, PA sequence analysis was the primary tool for baloxavir susceptibility assessment, as only two WHO CCs (Atlanta and Tokyo) had tested viruses phenotypically. To this end, PA sequences submitted by WHO CCs and other laboratories to GISAID were screened for amino acid substitutions associated with reduced susceptibility to this antiviral (PA marker table). Although not included in the WHO-AVWG table, residue 34 is part of the PA active site and one of the key residues (i.e., residues 20, 24, 34, 37, and 38) to which baloxavir binds (Omoto et al., 2018). Therefore, it was of interest to screen PA sequences for amino acid changes at this position. When isolates were available, flagged viruses were subjected to phenotypic testing to confirm the drug susceptibility phenotype. Also, phenotypic testing of viruses representing different subtypes/lineages was performed to calculate the median EC_50_ for each type/subtype/lineage.

For 2018–2019, a total of 15906 influenza viruses [7015 A(H1N1) pdm09, 7117 A(H3N2), 981 B/Victoria-lineage and 793 B/Yamagata-lineage viruses], representing 2.3% of virus detections on a global basis reported to FluNet, were primarily analyzed for the presence of substitutions associated with reduced baloxavir susceptibility using sequence-based analysis supplemented by phenotypic testing of those with known markers (where possible) and testing of a proportion lacking markers. The proportion of viruses analyzed varied by WHO region (AFRO: 3.2%; EMRO: 2.2%; EURO: 17.9%; PAHO: 55.6%; SEARO: 3.9%; WPRO: 17.2%).

**Fig. 4.**
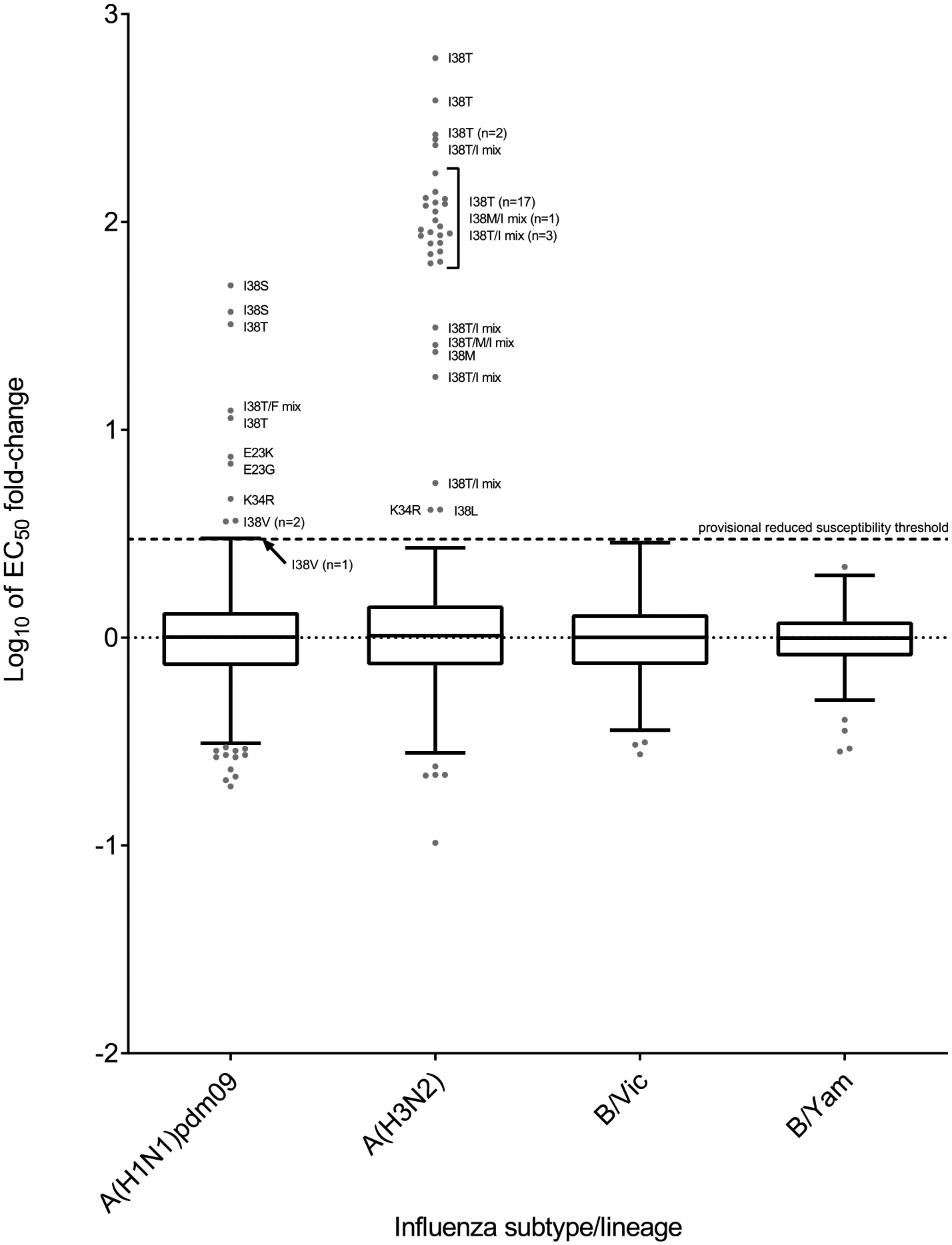
Column-scatter plots of log-transformed 50% effective concentration (EC_50_) fold-change values for the PAI baloxavir. The phenotypic susceptibility of influenza viruses to baloxavir was determined with cell culture–based assays, focus-reduction assay (FRA) or high-content imaging neutralization test (HINT). Overall, 1218 and 1110 viruses were tested phenotypically for 2018–2019 and 2019–2020, respectively. Data are presented by virus subtype or lineage [labelled on the x-axis: A(H1N1)pdm09; A(H3N2); B/Victoria-lineage; and B/Yamagata-lineage] and log-transformed EC_50_s on the y-axis. The boxes and whiskers are as defined in Fig. 3. An arbitrary cut-off of ≥3-fold increase from the median EC_50_ was used for reporting viruses with reduced susceptibility to baloxavir. Viruses without PA reduced susceptibility markers showed a <3-fold increase in EC_50_, as compared to the respective median EC_50_s for the two periods. PA markers were present in all viruses that showed a ≥3-fold increase in EC_50_, but not all viruses with markers showed ≥3-fold increases. PA amino acid substitutions are shown for viruses displaying reduced susceptibility. Amino acid position numbering is specific to type A and B viruses.

Most (9994/15505, 64.5%) PA sequences analyzed for this period were deposited in GISAID by three WHO CCs [Atlanta (n = 8911; 89.2%), Melbourne (n = 460; 4.6%), and Tokyo (n = 623; 6.2%)], with the remaining 5511 sequences (35.5%) submitted by other laboratories worldwide. A total of 72 PA sequences were flagged as containing previously reported (n = 68) or suspected (n = 4) reduced susceptibility markers; the latter were PA-K34R and PA-E23K + PA-K34Q (Table S6). A total of 1218 viruses from 2018–2019 were tested at the Atlanta CC(n = 387; 31.8%) or the Tokyo CC (n = 831; 68.2%). Baloxavir EC_50_s for the flagged viruses were determined and compared to the median EC_50_ to calculate a fold-change. The log_10_ transformed baloxavir EC_50_ fold-change values were used to prepare column-scatter plots, as was done for the NAI phenotypic data (Fig. 4). As expected, viruses without PA markers (n = 1172) showed a <3-fold increase when compared to the respective median EC_50_. PA marker substitutions were present in all viruses showing a ≥3-fold increase. However, five viruses with the markers PA-K34R, PA-I38F/I, PA-I38M/I, PA-I38V/I, or PA-E199G showed a <3-fold increase in EC_50_ as compared to the respective median EC_50_s (Table 3). Of these five viruses, two with PA-I38M/I and PA-I38V/I still showed a <3-fold increase in EC_50_ and the one with PA-E199G displayed a ≥3-fold increase when compared to the respective PA sequence-matched controls (Table S6).

For 2019–2020, a total of 15692 influenza viruses [5991 A(H1N1) pdm09, 4431 A(H3N2), 4935 B/Victoria-lineage and 335 B/Yamagata-lineage viruses], representing 2.3% of virus detections reported to FluNet were mainly analyzed for the presence of substitutions associated with reduced baloxavir susceptibility using sequence-based analysis supplemented by phenotypic testing. Most viruses (51.7%) were from the WHO PAHO region, followed by EURO (22.7%) and WPRO (16.7%) regions. The proportions for viruses from the WHO SEARO, AFRO, and EMRO regions were smaller (4.7%, 3.0%, and 1.2%, respectively).

Most (9821/15623, 62.9%) PA sequences were deposited by four WHO CCs [Atlanta (n = 7981; 81.3%), Beijing (n = 747; 7.6%), Melbourne (n = 279; 2.8%)] and Tokyo (n = 814; 8.3%)], with the remaining 5802 sequences (37.1%) submitted by other laboratories globally. The Atlanta and Tokyo CCs conducted phenotypic testing on 1110 viruses [Atlanta (n = 290; 26.1%), Tokyo (n = 820; 73.9%)], which included 7/18 PA sequence–flagged viruses (Fig. 4 and Table S6). All viruses that showed a ≥3-fold increase in EC_50_ had a PA reduced susceptibility marker (PA-I38T, PA-I38L, or PA-E23K), but not all viruses with other markers (PA-L28P, PA-M34I, or PA-I38V) showed a ≥3-fold increase relative to the subtype/lineage-specific median EC_50_ or the PA sequence–matched control virus EC_50_ (Tables 3 and S6).

Overall, combined sequence-based and phenotypic analysis revealed that the global frequency of influenza viruses with potentially reduced baloxavir susceptibility was low, being 0.5% (72/15906) and 0.1% (18/15692) for 2018–2019 and 2019–2020, respectively.

### 3.2. A(H1N1)pdm09 viruses

For the A(H1N1)pdm09 subtype, 21/7015 (0.3%) and 2/5991 (0.03%) viruses analyzed for 2018–2019 and 2019–2020, respectively, had PA reduced susceptibility markers (Table S6). Most (n = 18) of these viruses had PA-I38T/F/S/V substitutions, and substitutions at three other residues (23, 34, and 199) were detected. Notably, most viruses with a substitution at residue 38, except those with PA-I38V (n = 11), were collected from baloxavir-treated patients, and all but one (from Republic of Korea) were detected in Japan. Most variant viruses showed single amino acid substitutions at their reduced susceptibility marker positions, but a few with PA-I38 substitutions showed polymorphism (Table S6).

Six viruses that contained PA-I38F/S/T were tested and displayed 2.5- to 49.5-fold increases in EC_50_ compared to the median EC_50_. Only three viruses with PA-I38V were tested and they displayed 3.0- to 3.7-fold increases by FRA. The presence of valine at residue 38 represents a natural polymorphism and has not been linked to baloxavir treatment. Viruses with PA-E23G, PA-E23K (Takashita et al., 2020c) or PA-E199G showed 6.9-, 7.4- and 2.9-fold increases in EC_50,_ respectively (Table S6); the latter virus showed a 3.7-fold increase when compared to the PA sequence matched control. Two viruses (one each from Japan and the United States) had PA-K34R and showed 1.6- and 4.7-fold increases, respectively, in EC_50_ (Table S6). The reduced susceptibility of the United States virus was confirmed, with a 4.1-fold increase in the EC_50_, by comparing it to a PA sequence matched control.

### 3.3. A(H3N2) viruses

For the A(H3N2) subtype, 51/7117 (0.7%) and 8/4431 (0.2%) viruses analyzed for 2018–2019 and 2019–2020, respectively, had PA reduced susceptibility markers (Table S6). These variants were collected in 13 countries, but most were from Japan (n = 32) or the United States (n = 16). Of the 59 flagged viruses, 37 were available for phenotypic testing: 33 (including that with substitution at residue 34) displayed ≥3-fold increases in EC_50_ and four (with PA-L28P, PA-I38M/I or PA-I38V/I) exhibited <3-fold increases (Table S6).

Twenty-one viruses that contained PA-I38T alone showed 64.3- to 614.0-fold increases in EC_50_, and one virus with PA-I38T was not tested (Table 3). Eight viruses with mixtures at this position (PA-I38T/I or PA-I38T/M/I) showed 5.6- to 250.0-fold increases in EC_50_ (Tables 3 and S6). Two other viruses with PA-I38M or PA-I38M/I exhibited 23.7- to 91.8-fold increases in EC_50_. All these viruses were collected in Japan, and most were from baloxavir-treated patients <12 years of age (Table S6). The other flagged viruses had markers associated mainly with less common natural substitutions or polymorphisms: PA-I38L or PA-I38L/V/I (n = 2), PA-I38M or PA-I38M/I (n = 3), PA-I38V or PA-I38V/I (n = 3), and PA-L28P (n = 16) (Table S6). In addition, a virus from the Democratic Republic of the Congo had PA-K34R, one from the UK had PA-E23K + PA-K34Q, and one from Chile (Talca) had PA-E199G. Viruses with PA-I38L or PA-K34R showed 4.1-fold increases in EC_50_. All other available viruses showed fold-changes below the cut-off threshold, including two with PA-L28P. Notably, placing clinical specimens in culture led to the enrichment of virus isolates with PA-I38-substituted variants in several instances.

### 3.4. B/Victoria-lineage viruses

For B/Victoria-lineage viruses, 0/981 and 8/4935 (0.2%) viruses analyzed for 2018–2019 and 2019–2020, respectively, had PA reduced susceptibility markers (Table S6); the latter viruses were collected in five countries. Three viruses had PA-I38V, and the one tested showed <3-fold change in EC_50_. The remaining viruses contained PA-M34I or PA-M34V (Tables 3 and S6). The one PA-M34I virus tested showed a <3-fold increase in EC_50_ by FRA (Table S6).

### 3.5. B/Yamagata-lineage viruses

For B/Yamagata-lineage viruses, 0/793 and 0/335 viruses analyzed for 2018–2019 and 2019–2020, respectively, had PA reduced susceptibility markers.

## 4. Zoonotic viruses

Forty-nine cases of human infection with swine-lineage [A(H1N1) pdm09v, A(H1N2)v, A(H3N2)v subtypes] or avian-lineage [A(H5N1), A (H5N6), A(H7N9), A(H9N2) subtypes] influenza A viruses were reported for 2018–2019 and 2019–2020, including six cases of infection with highly pathogenic viruses (Table S7). The susceptibility of zoonotic influenza viruses to NAIs and baloxavir was assessed based on analysis of NA and PA sequences deposited in GISAID. Overall, sequences were available for 32 zoonotic viruses. Those viruses with an available NA sequence appear to be NAI susceptible, as no NA substitutions previously associated with RI/HRI phenotypes were found in them. A/Jiangsu/32888/2018 (H5N6) contained dual PA-I38V + PA-E199G substitutions. These substitutions alone have little or no effect on *in vitro* baloxavir susceptibility when present in other subtypes (PA marker table), but their combined effect has not been determined. Thirteen viruses had PA-A37S; this substitution did not alter baloxavir susceptibility in a recombinant A(H1N1) virus (Hashimoto et al., 2021). Of nine A(H1N2)v viruses collected in the United States during these two periods, three were tested for NAI susceptibility and six were tested for baloxavir susceptibility; all were susceptible to NAIs and baloxavir.

## 5. Concluding remarks

Several factors are thought likely to contribute to the detection rate of influenza viruses showing reduced susceptibility to antivirals. For example, when A(H3N2) viruses predominate, fewer viruses displaying RI/HRI by oseltamivir are typically detected. Conversely, higher detection rates are observed when A(H1N1)pdm09 viruses circulate widely; this is mainly due to oseltamivir-resistant NA-H275Y viruses. This potentially reflects the inherent abilities of N1 and N2 NAs to accommodate RI/HRI conferring substitutions without significantly affecting virus fitness (Collins et al., 2009).

The detection of A(H1N1)pdm09 viruses in untreated patients is an indicator of community transmission of resistant NA-H275Y viruses (Hurt et al., 2012; Takashita et al., 2015b), and this needs to be closely monitored. Notably, a small cluster of NA-H275Y viruses was detected in the United States in 2020 (Mohan et al., 2021) and the antigenically drifted hemagglutinin of these viruses may facilitate their spread.

Viruses with RI/HRI phenotypes can emerge as a result of natural amino acid polymorphism (sequence variance among circulating viruses). For example, natural polymorphism is the underlying factor in type B viruses displaying a RI/HRI phenotype to peramivir (Leang et al., 2014; Sleeman et al., 2011). Conversely, A(H3N2) viruses with a RI phenotype to zanamivir commonly emerge during virus culture, which promotes the selection of NA substitutions that reduce NA activity towards sialic acid–containing receptors. In addition, propagating viruses with a mixed population in culture can increase the proportion of variants with reduced susceptibility to antivirals; this applies to both NAIs and baloxavir.

It can be challenging to reconcile the outcomes of sequence-based analysis and phenotypic testing as both approaches have their limitations. For simplicity, the detection rate was calculated based on all markers listed in the WHO-AVWG tables, plus newly identified markers. However, not all markers conferred the expected drug phenotype. Another caveat is a border line NI/RI phenotype observed for viruses with certain markers. For example, NA-D197N was associated with NI/RI by zanamivir. Similarly, PA-L28P and PA-I38V conferred no change in baloxavir susceptibility. Such viruses can be counted as drug susceptible or displaying reduced susceptibility depending on the reference EC_50_ value used to calculate fold-change. As sequence-based analysis becomes a foundation for virologic surveillance, it would be beneficial to put additional efforts into establishing clear correlates between identified markers and drug susceptibility phenotype. To this end, recombinant viruses could be used to delineate the effect of a particular marker on a drug-susceptibility phenotype, or sequence-matched control viruses (e.g., PA sequence matched viruses) could be used for comparisons. This is especially important, because sequence-based analysis is the primary means of assessing the drug susceptibility of zoonotic influenza viruses as such viruses are often unavailable for phenotypic testing.

Overall, the proportions of viruses displaying reduced susceptibility to at least one antiviral were low (<1%) for both periods. However, during 2018–2019, there was a striking difference between Japan and the United States in the detection rate of PA reduced susceptibility markers. In Japan, the rate was 4.5% (41/919) and most of the detected viruses (n = 32) had PA-I38T, the principal treatment-emergent marker. Notably, most viruses with PA-I38T belonged to the A(H3N2) subtype and were isolated from young children with several patients not being exposed to baloxavir. This indicates the probable transmission of baloxavir-resistant viruses in local communities and/or households. It appears that using baloxavir to treat children <12 years of age may have contributed to the higher rate of baloxavir resistance in Japan in 2018–2019. In 2019, two Japanese medical professional societies issued recommendations to avoid prescribing baloxavir to children <12 years of age. In 2019–2020, the rate of baloxavir resistance detected in viruses collected in Japan was much lower at 0.4% (3/708) and only one virus, which was collected from a baloxavir-treated adult, had PA-I38T.

It is worth noting that in the United States, baloxavir was approved only for treating people aged 12 years or older. The detection rate was low in both periods: 0.3% (23/7024) in 2018–2019 and 0.1% (7/6509) in 2019–2020. None of the analyzed viruses had PA-I38T substitution. Also, samples submitted to the United States national surveillance program are typically collected before treatment is initiated. These factors may explain the low detection rate, which is consistent with the low circulation of PA variants with naturally reduced susceptibility to baloxavir (Gubareva et al., 2019).

Overall, the detection rate of viruses that either showed, or possessed markers predictive of reduced susceptibility to NAIs and/or the PAI baloxavir was low regardless of the usage of these antivirals. However, the data reported here indicate the need to continue the close monitoring and elucidation of factors contributing to reduced susceptibility to antivirals in influenza viruses.

## Supplementary Material

mmc1

mmc2

mmc3

## Figures and Tables

**Table 1:** Virus and patient characteristics for influenza type A viruses (n = 138) phenotypically tested by WHO CCs and showing reduced (RI) or highly reduced inhibition (HRI) by NAIs.^a^

Virus (n)	n	IC_50_ fold-change compared to reference median IC_50_ values^b^				NA substitution^c^		Patient setting	Antiviral treatment	Immunocompromised
		Oseltamivir	Zanamivir	Peramivir	Laninamivir	Virus isolate	Clinical specimen			
**A(H1N1) pdm09** (133 of 10280)	101	**286–2194**	0.5–3	**86–629** (75)	1–5 (75)	H275Y	Not available^d^ (30) H275Y (65) H275Y/H mix (6)	Community (42) Hospital (43) Unknown (16)	Yes, oseltamivir (20) Yes, peramivir (5) Yes, laninamivir (1) Yes, oseltamivir, baloxavir (1) Yes, peramivir, laninamivir (1) Yes, oseltamivir, laninamivir (3) No (29) Unknown (41)	Yes (5) No (62) Unknown (34)
	6	**24–976**	1–4 (4)	**32–197** (2)	3 (2)	H275Y/H mix	Not available (3) H275Y/H mix (2) None^e^ (1)	Community (1) Hospital (2) Unknown (3)	No (1) Unknown (5)	No (3) Unknown (3)
	1	**1376**	3	**746**	3	G147R/G mix, H275Y	Not available	Unknown	Unknown	Unknown
	10	0.5–2 (8)	**13–332**	**15–196** (5)	5–**56** (5)	Q136K (5) Q136K/Q mix, D151E/D mix (2) Q136K/Q mix, D151N/D mix (1) Q136R (2)	Not available (8) None (2)	Hospital (4) Unknown (5) Community (1)	Unknown (9) No (1)	Unknown (8) No (2)
	6	**11–28**	2–8	1–**11** (5)	1–5 (5)	I223K (3) I223L (1) I223M (1) I223R (1)	I223K (2) I223L (1) I223M (1) Not available (2)	Unknown (6)	Unknown (6)	Unknown (6)
	3	**96–191**	3–4	2–**13** (2)	3–4 (2)	N295S	Not available (1) N295S (2)	Unknown (2) Hospital (1)	Unknown (2) Yes (1)	Unknown (3)
	2	1–2	**26–81**	9–**83**	6–7	E119K (1) E119K, Q136K (1)	None	Hospital	Unknown	Unknown
	1	**11**	**11**	1	2	V116A	Not available	Unknown	Unknown	Unknown
	1	1	**321**	**24**	**58**	E119G	None	Hospital	Unknown	Unknown
	1	**52**	**21**	n/t^f^	n/t	R152K	Not available	Hospital	Unknown	Unknown
	1	**12**	3	2	1	D199G	None	Unknown	Unknown	Unknown
**A(H3N2)** (5 of 7755)	2	4.5–6	**12–21**	2 (1)	4 (1)	D151N/D mix (1) D151N/D mix, V165I (1)	Not available	Community (1) Sentinel outpatient (1)	Unknown (1) No (1)	Unknown (1) No (1)
	1	1	**17**	n/t	n/t	M241 V/M mix	Not available	Unknown	Yes	Unknown
	1	**15**	5	1.5	1	N245Y(-gly)	N245Y(-gly)	Hospital	Yes, peramivir	Unknown
	1	**11.5**	4	1	2	S331R	S331R	Community	No	No

a The number of viruses for which data were reported is shown in parentheses if it is less than the number in the ‘n’ column.

b Reduced inhibition (RI) and highly reduced inhibition (HRI) fold-change values are displayed underlined and in bold typeface. For type A viruses, normal inhibition (NI) is a <10-fold increase in the NAI IC_50_; RI is a 10- to 100-fold increase; and HRI is a >100-fold increase (World Health Organization, 2012).

c Amino acid position numbering is A subtype specific.

d Clinical specimen not available for sequencing.

e None: the sequence contained no amino acid substitutions when compared to the consensus sequence of viruses for the same subtype.

f n/t: not tested.

**Table 2:** Virus and patient characteristics for influenza type B viruses (n = 55) phenotypically tested by WHO CCs and showing reduced (RI) or highly reduced inhibition (HRI) by NAIs.^a^

Virus (n)	n	IC_50_ fold-change compared to reference median IC_50_ values^b^				NA substitution^c^		Patient setting	Antiviral treatment	Immunocompromised
		Oseltamivir Zanamivir		Peramivir	Laninamivir	Virus isolate	Clinical specimen			
**B/Victoria-lineage** (48 of 4701)	7	1–**10**	2–**78**	**5–1517**	2–**13**	E105K (5) E105K/E mix (2)	E105K/E mix (1) Not available^d^ (4) None^e^ (2)	Hospital (3) Unknown (4)	No (1) Unknown (6)	Unknown (7)
	5	2–4	4.5–**8**	3 (3)	3–4 (3)	D197N	D197N (2) Not available (2)	Hospital (2) Unknown (2)	Unknown (4)	Unknown (3) No (1)
	3	2–4	1–3	**6–32**	1–2	H134Y/H mix (3)	None (3)	Community (1) Unknown (2)	Unknown	Unknown (2) No (1)
	2	0.1–1	2–4	**6–7**	1–2	Q138K/Q mix (2)	None (2)	Community (1) Hospital (1)	No (1) Unknown (1)	No (1) Unknown (1)
	2	**6–7**	**12–41**	**99–259**	4.5–**6**	G140R	None (2)	Hospital (2)	Unknown (2)	Unknown (2)
	2	2–3	3–**9**	**5–6**	1–3	G145R	G145R (1) Not available (1)	Community (1) Emergency (1)	No (1) Unknown (1)	No (1) Unknown (1)
	2	0.5–**17**	**4–9**	**51–5763**	2	T146K	None (2)	Hospital (1) Community (1)	No (2)	Unknown (2)
	2	**5–7**	2–3	**7–10**	2	I221T (1) I221T/I mix (1)	I221T (1) None (1)	Hospital (1) Unknown (1)	Unknown (1) No (1)	Unknown (2)
	1	**5**	**55**	**829**	3	H101L	Not available	Unknown	Unknown	Unknown
	1	**6**	**5**	**18**	4	G108E	Not available	Hospital	Oseltamivir	Unknown
	1	3	3	**418**	2	T146P	None	Hospital	Unknown	Unknown
	1	**23**	**257**	n/t^f^	n/t	G243D	Not available	Hospital	Unknown	Unknown
	1	**19**	**22**	**58**	3	G243S	G243S	Unknown	Unknown	Unknown
	1	4	**15**	3	2	A245T	None	Hospital	No	Unknown
	1	1	1	**61**	1	H273Y	H273Y	Unknown	Unknown	Unknown
	1	3	**5**	n/t	n/t	I348V	Not available	Hospital	Unknown	Unknown
	1	1	2	52	1	D432G	Not available	Unknown	Unknown	Unknown
	1	0.4	**15**	**200**	3	H439P	Not available	Unknown	Unknown	Unknown
	1	0.1	2	**27**	1	H439R	None	Unknown	Unknown	Unknown
	1	4	3	**18**	2	T460I	None	Emergency	Unknown	Unknown
	1	2	4	**33**	2	S34L, K382R	Not available	Unknown	Unknown	Unknown
	1	2	2	**23**	2	H101L, K358R	K358R	Community	Unknown	Unknown
	1	1	1	**6**	2	G104R/G mix, E105K/E mix	None	Unknown	Unknown	Unknown
	1	1	**8**	**13**	3	G104R, G145R	None	Emergency	No	Unknown
	1	3	**5**	3	3	E105K, I115del	None	Emergency	Unknown	Unknown
	1	**5**	**64**	**348**	**30**	E105K, P139T/P mix	Not available	Unknown	Unknown	Unknown
	1	1	2	**5**	2	E105K, K382R	Not available	Unknown	Unknown	Unknown
	1	1	1	**18**	1	E105G, P139L	None	Emergency	Unknown	Unknown
	1	4	1	**243**	1	H134Y, D432G	None	Hospital	Unknown	Unknown
	1	**29**	**87**	**5707**	**6**	T146P, N169S	Not available	Unknown	Unknown	Unknown
	1	2	**9**	**103**	2	G247D, I361V	G247D, I361V	Hospital	Unknown	Unknown
**B/Yamagata-Lineage** (7 of 653)	2	0.3–**15**	0.5–1	**22–616**	1	H273Y (1) H273Y/H mix (1)	H273Y (1) H273Y/H mix (1)	Community (1) Unknown (1)	No (1) Unknown (1)	No (1) Unknown (1)
	1	**5**	3	3	4	D197N	D197N	Unknown	Unknown	Unknown
	1	**7**	3	**9**	1	A200T	A200T	Hospital	Unknown	Unknown
	1	1	1	**7**	2	I221V	I221V	Unknown	Unknown	Unknown
	1	1	**12**	2	3	S249N	Not available	Unknown	Unknown	Unknown
	1	**8**	**5**	**13**	4	No mutation detected	Not sequenced	Community	Unknown	Unknown

a The number of viruses for which data were reported is shown in parentheses if it is less than the number in the ‘n’ column.

b Reduced inhibition (RI) and highly reduced inhibition (HRI) fold-change values are displayed underlined and in bold typeface. For type B viruses, normal inhibition (NI) is a <5-fold increase in the NAI IC_50_; RI is a 5- to 50-fold increase; and HRI is a >50-fold increase (World Health Organization, 2012).

c Amino acid position numbering is B-type specific.

d Clinical specimen not available for testing.

e None: the virus contained no amino acid substitutions when compared to viruses with a normal inhibition (NI) phenotype.

f n/t: not tested.

**Table 3:** Virus and patient characteristics for type A and B influenza viruses (n = 53) containing PA amino acid substitutions of concern and phenotypically tested by WHO CCs for baloxavir susceptibility.^a^

Virus (n)	n	EC_50_ fold-change as compared to reference median EC_50_ values	PA substitution^b^		Patient setting	Antiviral treatment	Immunocompromised
			Virus isolate	Clinical specimen			
**A(H1N1)pdm09** (14 of 984)	2	11.4–32.2	I38T	I38T/I mix	Community	Yes, baloxavir	No
	1	12.4	I38 T/F mix	I38T/F/I mix	Community	Yes, baloxavir	No
	2	36.9–49.5	I38S	I38S	Community	Yes, baloxavir	No
	3	3.0–3.7	I38V	I38V	Community	Yes, laninamivir	No
	1	2.5	I38F/I mix	I38F/I mix	Unknown	Unknown	Unknown
	1	6.9	E23G	E23G	Unknown	Unknown	Unknown
	1	7.4	E23K	E23K	Community	No	No
	2	1.6–4.7	K34R	K34R	Unknown	Unknown	Unknown
	1	2.9	E199G	E199G	Unknown	Unknown	Unknown
**A(H3N2)** (37 of 768)	21	64.3–614.0	I38T	I38T (10) I38T/I mix (9) I38T/K/I mix (1) Not available^c^ (1)	Community (19) Hospital (2)	Yes, baloxavir (17) Yes, oseltamivir (1) No (3)	No (20) Unknown (1)
	7	5.6–250.0	I38T/I mix	I38T/I mix	Community	Yes, baloxavir	No
	1	25.6	I38 T/M/I mix	I38T/M/I mix	Community	Yes, baloxavir	No
	1	23.7	I38M	I38M	Community	Yes, baloxavir	No
	2	1.0–91.8	I38M/I mix	I38M/I mix (1) I38 (1)	Community (1) Unknown (1)	Yes, baloxavir (1) Unknown (1)	No (1) Unknown (1)
	1	4.1	I38L	I38L	Unknown	Unknown	Unknown
	1	4.1	K34R	K34R	Unknown	Unknown	Unknown
	2	0.5–1.3	L28P	L28P (1) Not available (1)	Unknown	Unknown	Unknown
	1	0.9	I38V/I mix	Not available	Unknown	Unknown	Unknown
**B/Victoria** (2 of 425)	1	0.9	I38V	I38V	Unknown	Unknown	Unknown
	1	0.6	M34I	Not available	Unknown	Unknown	Unknown

a The number of viruses for which data were reported is shown in parentheses if the number is less than the number in the ‘n’ column.

b PA amino acid substitutions associated with reduced susceptibility to baloxavir, as listed in the summary table provided by the AVWG on the WHO website (https://cdn.who.int/media/docs/default-source/influenza/summary-of-polymerase-acidic-(pa)-protein-amino-acid-substitutions-analysed-for-their-effects-on-baloxavir-susceptibility.pdf?sfvrsn=442806e_1&download=true) are shown. Substitutions at PA residue 34 are included.

c Clinical specimen not available for testing.

## References

[R1] Air GM, Els MC, Brown LE, Laver WG, Webster RG, 1985. Location of antigenic sites on the three-dimensional structure of the influenza N2 virus neuraminidase. Virol 145 (2), 237–248. 10.1016/0042-6822(85)90157-6 2411049

[R2] Beigel JH, Hayden FG, 2021. Influenza therapeutics in clinical practice—challenges and recent advances. Cold Spring Harb. Perspect. Med 11 (4), a038463. 32041763 10.1101/cshperspect.a038463 PMC8015700

[R3] Boltz DA, Douangngeun B, Phommachanh P, Sinthasak S, Mondry R, Obert C, Seiler P, Keating R, Suzuki Y, Hiramatsu H, Govorkova EA, Webster RG, 2010. Emergence of H5N1 avian influenza viruses with reduced sensitivity to neuraminidase inhibitors and novel reassortants in Lao People’s Democratic Republic. J. Gen. Virol 91 (4), 949–959. 20016036 10.1099/vir.0.017459-0 PMC2888158

[R4] Collins PJ, Haire LF, Lin YP, Liu J, Russell RJ, Walker PA, Martin SR, Daniels RS, Gregory V, Skehel JJ, Gamblin SJ, Hay AJ, 2009. Structural basis for oseltamivir resistance of influenza viruses. Vaccine 27 (45), 6317–6323. 19840667 10.1016/j.vaccine.2009.07.017

[R5] Dharan NJ, Gubareva LV, Meyer JJ, Okomo-Adhiambo M, McClinton RC, Marshall SA, St George K, Epperson S, Brammer L, Klimov AI, Bresee JS, Fry AM, Oseltamivir-Resistance Working Group., 2009. Infections with oseltamivir-resistant influenza A(H1N1) virus in the United States. JAMA 301 (10), 1034–1041. 19255110 10.1001/jama.2009.294

[R6] Gubareva LV, Besselaar TG, Daniels RS, Fry A, Gregory V, Huang W, Hurt AC, Jorquera PA, Lackenby A, Leang SK, Lo J, Pereyaslov D, Rebelo-de-Andrade H, Siqueira MM, Takashita E, Odagiri T, Wang D, Zhang W, Meijer A, 2017. Global update on the susceptibility of human influenza viruses to neuraminidase inhibitors, 2015–2016. Antivir. Res 146, 12–20. 28802866 10.1016/j.antiviral.2017.08.004 PMC5667636

[R7] Gubareva LV, Mishin VP, Patel MC, Chesnokov A, Nguyen HT, De La Cruz J, Spencer S, Campbell AP, Sinner M, Reid H, Garten R, Katz JM, Fry AM, Barnes J, Wentworth DE, 2019. Assessing baloxavir susceptibility of influenza viruses circulating in the United States during the 2016/17 and 2017/18 seasons. Euro Surveill 24 (3), 1800666. 10.2807/1560-7917.ES.2019.24.3.1800666 PMC6344838 30670144

[R8] Hashimoto T, Baba K, Inoue K, Okane M, Hata S, Shishido T, Naito A, Wildum S, Omoto S, 2021. Comprehensive assessment of amino acid substitutions in the trimeric RNA polymerase complex of influenza A virus detected in clinical trials of baloxavir marboxil. Influenza Other Respir. Viruses 15 (3), 389–395. 33099886 10.1111/irv.12821 PMC8051730

[R9] Hayden FG, Sugaya N, Hirotsu N, Lee N, de Jong MD, Hurt AC, Ishida T, Sekino H, Yamada K, Portsmouth S, Kawaguchi K, Shishido T, Arai M, Tsuchiya K, Uehara T, Watanabe A, for the Baloxavir Marboxil Investigators Group, 2018. Baloxavir marboxil for uncomplicated influenza in adults and adolescents. N. Engl. J. Med 379 (10), 913–923. 30184455 10.1056/NEJMoa1716197

[R10] Hurt AC, Selleck P, Komadina N, Shaw R, Brown L, Barr IG, 2007. Susceptibility of highly pathogenic A(H5N1) avian influenza viruses to the neuraminidase inhibitors and adamantanes. Antivir. Res 73 (3), 228–231. 17112602 10.1016/j.antiviral.2006.10.004

[R11] Hurt AC, Ernest J, Deng YM, Iannello P, Besselaar TG, Birch C, Buchy P, Chittaganpitch M, Chiu SC, Dwyer D, Guigon A, Harrower B, Kei IP, Kok T, Lin C, McPhie K, Mohd A, Olveda R, Panayotou T, Rawlinson W, Scott L, Smith D, D’Souza H, Komadina N, Shaw R, Kelso A, Barr IG, 2009. Emergence and spread of oseltamivir-resistant A(H1N1) influenza viruses in Oceania, South East Asia and South Africa. Antivir. Res 83 (1), 90–93. 19501261 10.1016/j.antiviral.2009.03.003

[R12] Hurt AC, Hardie K, Wilson NJ, Deng YM, Osbourn M, Leang SK, Lee RTC, Iannello P, Gehrig N, Shaw R, Wark P, Caldwell N, Givney RC, Xue L, Maurer-Stroh S, Dwyer DE, Wang B, Smith DW, Levy A, Booy R, Dixit R, Merritt T, Kelso A, Dalton C, Durrheim D, Barr IG, 2012. Characteristics of a widespread community cluster of H275Y oseltamivir-resistant A(H1N1)pdm09 influenza in Australia. J. Infect. Dis 206 (2), 148–157. 22561367 10.1093/infdis/jis337 PMC3379839

[R13] Hurt AC, Besselaar TG, Daniels RS, Ermetal B, Fry A, Gubareva L, Huang W, Lackenby A, Lee RT, Lo J, Maurer-Stroh S, Nguyen HT, Pereyaslov D, Rebelo-de-Andrade H, Siqueira MM, Takashita E, Tashiro M, Tilmanis D, Wang D, Zhang W, Meijer A, 2016. Global update on the susceptibility of human influenza viruses to neuraminidase inhibitors, 2014–2015. Antivir. Res 132, 178–185. 27265623 10.1016/j.antiviral.2016.06.001 PMC5357725

[R14] Hussain S, Daniels RS, Wharton SA, Howell S, Halai C, Kunzelmann S, Whittaker L, McCauley JW, 2021. Reduced sialidase activity of influenza A (H3N2) neuraminidase associated with positively charged amino acid substitutions. J. Gen. Virol 102 (10). 10.1099/jgv.0.001648 34596510

[R15] Imai M, Yamashita M, Sakai-Tagawa S, Iwatsuki-Horimoto K, Kiso M, Murakami J, Yasuhara A, Takada K, Ito M, Nakajima N, Takahashi K, Lopes TJS, Dutta J, Khan Z, Kriti D, van Bakel H, Tokita A, Hagiwara H, Izumida N, Kuroki H, Nishino T, Wada N, Koga M, Adachi E, Jubishi D, Hasegawa H, Kawaoka Y, 2020. Influenza A variants with reduced susceptibility to baloxavir isolated from Japanese patients are fit and transmit through respiratory droplets. Nat. Microbiol 5 (1), 27–33. 31768027 10.1038/s41564-019-0609-0 PMC13014278

[R16] Ince WL, Smith FB, O’Rear JJ, Thomson M, 2020. Treatment-emergent influenza virus polymerase acidic substitutions independent of those at I38 associated with reduced baloxavir susceptibility and virus rebound in trials of baloxavir marboxil. J. Infect. Dis 222 (6), 957–961. 32253432 10.1093/infdis/jiaa164

[R17] Ison MG, Portsmouth S, Yoshida Y, Shishido T, Mitchener M, Tsuchiya K, Uehara T, Hayden FG, 2020. Early treatment with baloxavir marboxil in high-risk adolescent and adult outpatients with uncomplicated influenza (CAPSTONE-2): a randomised, placebo-controlled, phase 3 trial. Lancet Infect. Dis 20 (10), 1204–1214. 32526195 10.1016/S1473-3099(20)30004-9

[R18] Ison MG, Hayden FG, Hay AJ, Gubareva LV, Govorkova EA, Takashita E, McKimm-Breschkin JL, 2021. Influenza polymerase inhibitor resistance: assessment of the current state of the art—a report of the isirv Antiviral group. Antivir. Res 194, 105158. 34363859 10.1016/j.antiviral.2021.105158 PMC9012257

[R19] Lackenby A, Besselaar TG, Daniels RS, Fry A, Gregory V, Gubareva LV, Huang W, Hurt AC, Leang SK, Lee RTC, Lo J, Lollis L, Maurer-Stroh S, Odagiri T, Pereyaslov D, Takashita E, Wang D, Zhang W, Meijer A, 2018. Global update on the susceptibility of human influenza viruses to neuraminidase inhibitors and status of novel antivirals, 2016–2017. Antivir. Res 157, 38–46. 29981793 10.1016/j.antiviral.2018.07.001 PMC6094047

[R20] Leang SK, Kwok S, Sullivan SG, Maurer-Stroh S, Kelso A, Barr IG, Hurt AC, 2014. Peramivir and laninamivir susceptibility of circulating influenza A and B viruses. Influenza Other Respir. Viruses 8 (2), 135–139. 24734292 10.1111/irv.12187 PMC4186459

[R21] Lee N, Hurt AC, 2018. Neuraminidase inhibitor resistance in influenza: a clinical perspective. Curr. Opin. Infect. Dis 31 (6), 520–526. 30299356 10.1097/QCO.0000000000000498

[R22] Little K, Leang S-K, Butler J, Baas C, Harrower B, Mosse J, Barr IG, Hurt AC, 2015. Zanamivir-resistant influenza viruses with Q136K or Q136R neuraminidase residue mutations can arise during MDCK cell culture creating challenges for antiviral susceptibility monitoring. Euro Surveill 20 (45), 30060. 10.2807/1560-7917.ES.2015.20.45.30060 26608955

[R23] Meijer A, Lackenby A, Hungnes O, Lina B, van-der-Werf S, Schweiger B, Opp M, Paget J, van-de-Kassteele J, Hay A, Zambon M, European Influenza Surveillance Scheme, 2009. Oseltamivir-resistant influenza virus A (H1N1), Europe, 2007–08 season. Emerg. Infect. Dis 15 (4), 552–560. 19331731 10.3201/eid1504.081280 PMC2671453

[R24] Meijer A, Rebelo-de-Andrade H, Correia V, Besselaar T, Drager-Dayal R, Fry A, Gregory V, Gubareva L, Kageyama T, Lackenby A, Lo J, Odagiri T, Pereyaslov D, Siqueira MM, Takashita E, Tashiro M, Wang D, Wong S, Zhang W, Daniels RS, Hurt AC, 2014. Global update on the susceptibility of human influenza viruses to neuraminidase inhibitors, 2012–2013. Antivir. Res 110, 31–41. 25043638 10.1016/j.antiviral.2014.07.001 PMC8851378

[R25] Mohan T, Nguyen HT, Kniss K, Mishin VP, Merced-Morales AA, Laplante J, St George K, Blevins P, Chesnokov A, De La Cruz JA, Kondor R, Wentworth DE, Gubareva LV, 2021. Cluster of oseltamivir-resistant and hemagglutinin antigenically drifted influenza A(H1N1)pdm09 viruses, Texas, USA, January 2020. Emerg. Infect. Dis 27 (7), 1953–1957. 34152954 10.3201/eid2707.204593 PMC8237887

[R26] Omoto S, Speranzini V, Hashimoto T, Noshi T, Yamaguchi H, Kawai M, Kawaguchi K, Uehara T, Shishido T, Naito A, Cusack S, 2018. Characterization of influenza virus variants induced by treatment with the endonuclease inhibitor baloxavir marboxil. Sci. Rep 8 (1), 9633. 29941893 10.1038/s41598-018-27890-4 PMC6018108

[R27] Sleeman K, Sheu TG, Moore Z, Kilpatrick S, Garg S, Fry AM, Gubareva LV, 2011. Influenza B viruses with mutation in the neuraminidase active site, North Carolina, USA, 2010–11. Emerg. Infect. Dis 17 (11), 2043–2046. 22099093 10.3201/eid1711.110787 PMC3310577

[R28] Takada K, Kawakami C, Fan S, Chiba S, Zhong G, Gu C, Shimizu K, Takasaki S, Sakai-Tagawa Y, Lopes TJS, Dutta J, Khan Z, Kriti D, van Bakel H, Yamada S, Watanabe T, Imai M, Kawaoka Y, 2019. A humanized MDCK cell line for the efficient isolation and propagation of human influenza viruses. Nat. Microbiol 4 (8), 1268–1273. 31036910 10.1038/s41564-019-0433-6 PMC12421904

[R29] Takashita E, Meijer A, Lackenby A, Gubareva L, Rebelo-de-Andrade H, Besselaar T, Fry A, Gregory V, Leang SK, Huang W, Lo J, Pereyaslov D, Siqueira MM, Wang D, Mak GC, Zhang W, Daniels RS, Hurt AC, Tashiro M, 2015a. Global update on the susceptibility of human influenza viruses to neuraminidase inhibitors, 2013–2014. Antivir. Res 117, 27–38. 25721488 10.1016/j.antiviral.2015.02.003 PMC9036627

[R30] Takashita E, Kiso M, Fujisaki S, Yokoyama M, Nakamura K, Shirakura M, Sato H, Odagiri T, Kawaoka Y, Tashiro M, 2015b. Characterization of a large cluster of influenza A(H1N1)pdm09 viruses cross-resistant to oseltamivir and peramivir during the 2013–2014 influenza season in Japan. Antimicrob. Agents Chemother 59 (5), 2607–2617. 25691635 10.1128/AAC.04836-14 PMC4394804

[R31] Takashita E, Fujisaki S, Shirakura M, Nakamura K, Kishida N, Kuwahara T, Shimazu Y, Shimomura T, Watanabe S, Odagiri T, Influenza Virus Surveillance Group of Japan, 2016. Influenza A(H1N1)pdm09 virus exhibiting enhanced cross-resistance to oseltamivir and peramivir due to a dual H275Y/G147R substitution, Japan, March 2016. Euro Surveill 21 (24), 30258. 10.2807/1560-7917.ES.2016.21.24.30258 27336226

[R32] Takashita E, Morita H, Ogawa R, Nakamura K, Fujisaki S, Shirakura M, Kuwahara T, Kishida N, Watanabe S, Odagiri T, 2018. Susceptibility of influenza viruses to the novel cap-dependent endonuclease inhibitor baloxavir marboxil. Front. Microbiol 9, 3026. 30574137 10.3389/fmicb.2018.03026 PMC6291754

[R33] Takashita E, Daniels RS, Fujisaki S, Gregory V, Gubareva LV, Huang W, Hurt AC, Lackenby A, Nguyen HT, Pereyaslov D, Roe M, Samaan M, Subbarao K, Tse H, Wang D, Yen H-L, Zhang W, Meijer A, 2020a. Global update on the susceptibilities of human influenza viruses to neuraminidase inhibitors and the cap-dependent endonuclease inhibitor baloxavir, 2017–2018. Antivir. Res 175, 104718. 32004620 10.1016/j.antiviral.2020.104718

[R34] Takashita E, Yasui Y, Nagata S, Morita H, Fujisaki S, Miura H, Shirakura M, Kishida N, Nakamura K, Kuwahara T, Sugawara H, Sato A, Akimoto M, Kaido T, Watanabe S, Hasegawa H, The Influenza Virus Surveillance Group of Japan, 2020b. Detection of a peramivir-resistant influenza B/Yamagata-lineage virus imported from Indonesia in Aichi, Japan, March 2019. Jpn. J. Infect. Dis 73, 386–390. 32475875 10.7883/yoken.JJID.2020.084

[R35] Takashita E, Abe T, Morita H, Nagata S, Fujisaki S, Miura H, Shirakura M, Kishida N, Nakamura K, Kuwahara T, Mitamura K, Ichikawa M, Yamazaki M, Watanabe S, Hasegawa H, Influenza Virus Surveillance Group of Japan, 2020c. Influenza A(H1N1)pdm09 virus exhibiting reduced susceptibility to baloxavir due to a PA E23K substitution detected from a child without baloxavir treatment. Antivir. Res 180, 104828. 32574689 10.1016/j.antiviral.2020.104828

[R36] Uehara T, Hayden FG, Kawaguchi K, Omoto S, Hurt AC, De Jong MD, Hirotsu N, Sugaya N, Lee N, Baba K, Shishido T, Tsuchiya K, Portsmouth S, Kida H, 2020. Treatment-emergent influenza variant viruses with reduced baloxavir susceptibility: impact on clinical and virologic outcomes in uncomplicated influenza. J. Infect. Dis 221 (3), 346–355. 31309975 10.1093/infdis/jiz244

[R37] World Health Organization, 2012. Meetings of the WHO working group on surveillance of influenza antiviral susceptibility—Geneva, November 2011 and June 2012. Wkly. Epidemiol. Rec 87 (39), 369–374. 23061103

