# Supplementary material for: Global update on the susceptibilities of human influenza viruses to neuraminidase inhibitors and the cap-dependent endonuclease inhibitor baloxavir, 2018–2020
Source: Antiviral Res. Author manuscript; Available in PMC 2022 Jul 5. (PMC9254721; doi:10.1016/j.antiviral.2022.105281)
Supplement: mmc1 [file NIHMS1791090-supplement-mmc1.pptx]

## Slide 1
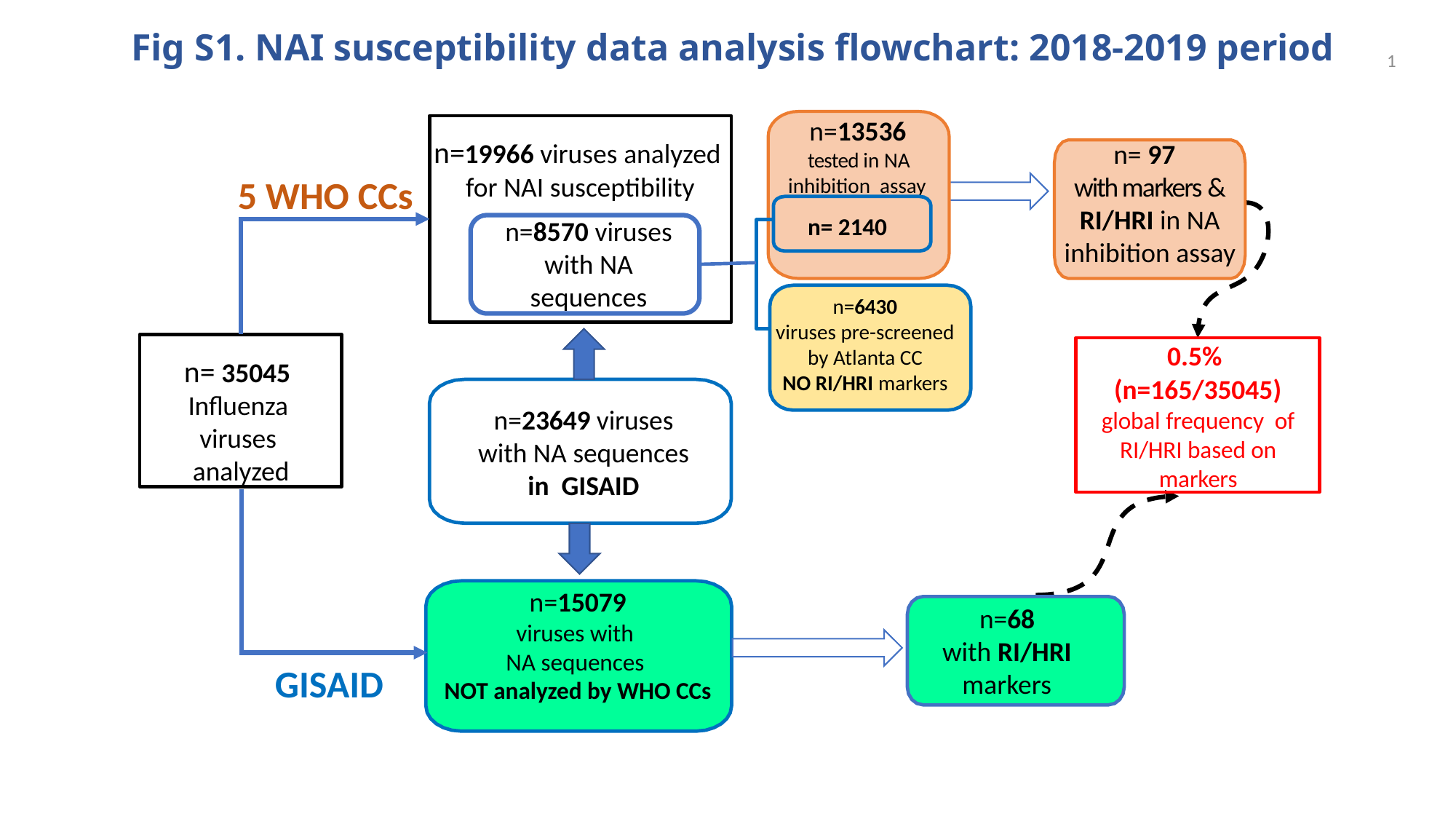

Fig S1. NAI susceptibility data analysis flowchart: 2018-2019 period
1
n=13536
tested in NA inhibition assay
n=19966 viruses analyzed
for NAI susceptibility
n= 97
with markers &
RI/HRI in NA inhibition assay
5 WHO CCs
n=8570 viruses with NA sequences
n= 2140
n=6430
viruses pre-screened by Atlanta CC
NO RI/HRI markers
n= 35045
Influenza viruses analyzed
0.5%
(n=165/35045)
global frequency of RI/HRI based on markers
n=23649 viruses
with NA sequences in GISAID
n=15079
viruses with
NA sequences
NOT analyzed by WHO CCs
n=68
with RI/HRI markers
GISAID

## Slide 2
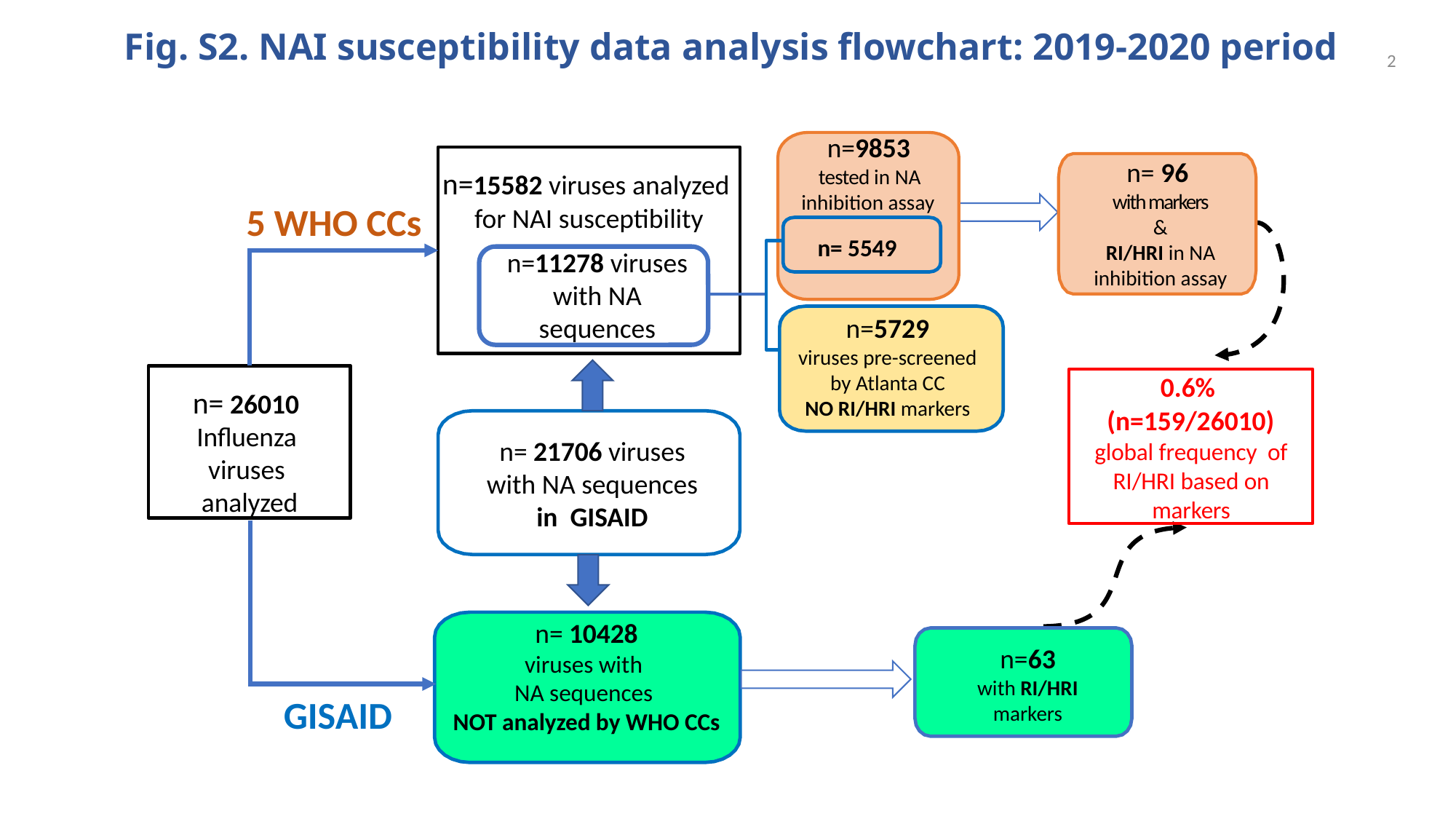

Fig. S2. NAI susceptibility data analysis flowchart: 2019-2020 period
2
n=9853
tested in NA inhibition assay
n=15582 viruses analyzed
for NAI susceptibility
n= 96
with markers
&
RI/HRI in NA inhibition assay
5 WHO CCs
n= 5549
n=11278 viruses with NA sequences
n=5729
viruses pre-screened by Atlanta CC
NO RI/HRI markers
n= 26010
Influenza viruses analyzed
0.6%
(n=159/26010)
global frequency of RI/HRI based on markers
n= 21706 viruses
with NA sequences in GISAID
n= 10428
viruses with
NA sequences
NOT analyzed by WHO CCs
n=63
with RI/HRI markers
GISAID
