# Supplementary material for: Global update on the susceptibilities of human influenza viruses to neuraminidase inhibitors and the cap-dependent endonuclease inhibitor baloxavir, 2018–2020
Source: Antiviral Res. Author manuscript; Available in PMC 2022 Jul 5. (PMC9254721; doi:10.1016/j.antiviral.2022.105281)
Supplement: mmc2 [file NIHMS1791090-supplement-mmc2.docx]

**Supplementary Figure Legends**

**Fig. S1.** NAI susceptibility data analysis flowchart: 2018-2019 period. During this period, a total of 35045 viruses (five WHO CCs + NA sequences from GISAID) were assessed to determine the global frequency of influenza viruses exhibiting or expected to exhibit RI/HRI using phenotypic and/or sequence-based methods. Among this, five WHO CCs together assessed NAI susceptibility of 19966 influenza viruses. Of this, 13536 viruses were tested phenotypically by NA inhibition assays, and potential NAI susceptibility of 6430 viruses were assessed by Atlanta CC based on NA sequence analysis. Overall, phenotypic testing identified 97/13536 viruses with RI/HRI. Sequence analysis of 6430 viruses identified no NA substitutions associated with RI/HRI phenotypes. At the other hand, NA sequences of 23649 viruses collected during 2018–2019 period were deposited in the GISAID. According to strain designation, 8570 sequences belonged to viruses submitted by five WHO CCs for NAI susceptibility analysis. For the remaining 15079 viruses, NA sequences were analyzed for the presence of substitutions previously associated with RI/HRI. A total of 68 viruses were identified with NA substitutions associated with RI/HRI. Thus, global frequency of influenza viruses displaying RI/HRI or with potential to exhibit RI/HRI by NAIs was 0.5% (165/35045) for 2018-2019 period.

**Fig. S2.** NAI susceptibility data analysis flowchart: 2019-2020 period. During this period, a total of 26010 viruses (five WHO CCs + NA sequences from GISAID) were assessed to determine the global frequency of influenza viruses exhibiting or expected to exhibit RI/HRI using phenotypic and/or sequence-based methods. Among this, five WHO CCs together assessed NAI susceptibility of 15582 influenza viruses. Of this, 9853 viruses were tested phenotypically by NA inhibition assays, and potential NAI susceptibility of 5729 viruses were assessed by Atlanta CC based on NA sequence analysis. Overall, phenotypic testing identified 96/9853 viruses with RI/HRI. Sequence analysis of 5729 viruses identified no NA substitutions associated with RI/HRI phenotypes. At the other hand, NA sequences of 21706 viruses collected during 2019–2020 period were deposited in the GISAID. According to strain designation, 11278 sequences belonged to viruses submitted by five WHO CCs for NAI susceptibility analysis. For the remaining 10428 viruses, NA sequences were analyzed for the presence of substitutions previously associated with RI/HRI. A total of 63 viruses were identified with NA substitutions associated with RI/HRI. Thus, global frequency of influenza viruses displaying RI/HRI or with potential to exhibit RI/HRI by NAIs was 0.6% (159/26010) for 2019-2020 period.
