# Supplementary material for: Global update on the susceptibilities of human influenza viruses to neuraminidase inhibitors and the cap-dependent endonuclease inhibitor baloxavir, 2018–2020
Source: Antiviral Res. Author manuscript; Available in PMC 2022 Jul 5. (PMC9254721; doi:10.1016/j.antiviral.2022.105281)
Supplement: mmc3 [file NIHMS1791090-supplement-mmc3.docx]

**Table S1.** Supply of influenza antiviral to medical institutes in Japan, 2018-2019 and 2019-2020 periods.

| **Antiviral** | **Supply doses (x10,000)** | |
| --- | --- | --- |
|  | **2018-2019**^a^ | **2019-2020** |
| Zanamivir | 59 | 50 |
| Oseltamivir | 464 | 336 |
| Peramivir | 32 | 19 |
| Laninamivir | 289 | 266 |
| Baloxavir | 528 | 60 |
| Total | 1372 | 731 |

^a^ Influenza antiviral supply to medical institutions in Japan for 2018-2019 was published in a review by Takashita, E., 2021. Influenza polymerase inhibitors: mechanisms of action and resistance. Cold Spring Harb. Perspect. Med. 11(5): a038687.

**Table S2.** The potential NAI susceptibility of influenza viruses based on NA sequence analysis assessed by Atlanta CC.

| **Source of virus isolation** | **No. of NA sequences analyzed**  **by Atlanta CC/Total no. of NA sequences** **analyzed (%)** | **Geographical area of**  **virus isolation (n)** |
| --- | --- | --- |
| **2018-2019 period** | | |
| US | 2916/6430 (45%) | Americas (1) |
| Non-US | 3514/6430 (55%) | Africa (13), Americas (35), Eastern Mediterranean (7), Europe (7), South-Ease Asia (5), Western Pacific (7) |
| **2019-2020 period** | | |
| US | 2919/5729 (51%) | Americas (1) |
| Non-US | 2810/5729 (49%) | Africa (9), Americas (37), Eastern Mediterranean (6), Europe (5), South-Ease Asia (4), Western Pacific (4) |

**Table S3.** Influenza A(H1N1)pdm09 and A(H3N2) virus isolates showing RI or HRI by one or more NAIs (n = 138).

| # | Type/subtype | Strain designation | WHO CC | IC_50_ fold-change as compared to reference  median IC_50_ values^a^ | | | | Substitution in virus isolate^b^ | Substitution in original specimen^b^ | Patient  setting | Antiviral  treatment | Immuno-compromised | Country of specimen collection | Date of collection (y/m/d) |
| --- | --- | --- | --- | --- | --- | --- | --- | --- | --- | --- | --- | --- | --- | --- |
|  |  |  |  | Oseltamivir | Zanamivir | Peramivir | Laninamivir |  |  |  |  |  |  |  |
| 1 | A(H1N1)pdm09 | A/Bolivia/245/2020 | Atlanta | **1057** | 1 | **278** | 2 | H275Y | H275Y | Unknown | Unknown | Unknown | Bolivia | 2020/03/03 |
| 2 | A(H1N1)pdm09 | A/Texas/26/2020 | Atlanta | **1093** | 2 | **346** | 2 | H275Y | H275Y | Unknown | Unknown | Unknown | United States | 2020/01/24 |
| 3 | A(H1N1)pdm09 | A/Michigan/15/2020 | Atlanta | **1041** | 2 | **156** | 2 | H275Y | H275Y | Unknown | Unknown | Unknown | United States | 2020/01/04 |
| 4 | A(H1N1)pdm09 | A/Utah/49/2019 | Atlanta | **736** | 1 | **158** | 2 | H275Y | H275Y | Unknown | Unknown | Unknown | United States | 2019/10/25 |
| 5 | A(H1N1)pdm09 | A/Taiwan/82637/2019 | Atlanta | **729** | 1 | **86** | 3 | H275Y | Not available^c^ | Unknown | Unknown | Unknown | Taiwan | 2019/07/08 |
| 6 | A(H1N1)pdm09 | A/Florida/61/2019 | Atlanta | **735** | 1 | **204** | 3 | H275Y | H275Y | Unknown | Unknown | Unknown | United States | 2019/04/29 |
| 7 | A(H1N1)pdm09 | A/Connecticut/30/2019 | Atlanta | **824** | 1 | **334** | 3 | H275Y | H275Y | Unknown | Unknown | Unknown | United States | 2019/03/31 |
| 8 | A(H1N1)pdm09 | A/Texas/110/2019 | Atlanta | **833** | 2 | **273** | 2 | H275Y | H275Y | Unknown | No | Unknown | United States | 2019/02/11 |
| 9 | A(H1N1)pdm09 | A/Alabama/03/2020 | Atlanta | **657** | 1 | **138** | 2 | H275Y | H275Y | Unknown | Unknown | Unknown | United States | 2019/02/04 |
| 10 | A(H1N1)pdm09 | A/St Kitts/2299/2019 | Atlanta | **648** | 1 | **222** | 3 | H275Y | Not available | Unknown | Unknown | Unknown | St Kitts | 2019/01/26 |
| 11 | A(H1N1)pdm09 | A/New York/58/2018 | Atlanta | **466** | 1 | **257** | 2 | H275Y | H275Y | Unknown | No | Unknown | United States | 2018/12/24 |
| 12 | A(H1N1)pdm09 | A/California/107/2018 | Atlanta | **823** | 1 | **261** | 2 | H275Y | H275Y | Unknown | Yes, oseltamivir | Unknown | United States | 2018/12/09 |
| 13 | A(H1N1)pdm09 | A/Hong Kong/2272/2018 | Atlanta | **612** | 2 | **236** | 3 | H275Y | H275Y | Unknown | Unknown | Unknown | Hong Kong | 2018/10/05 |
| 14 | A(H1N1)pdm09 | A/Mexico/2471/2018 | Atlanta | **780** | 1 | **212** | 2 | H275Y | Not available | Unknown | Unknown | Unknown | Mexico | 2018/09/14 |
| 15 | A(H1N1)pdm09 | A/Panama/324954/2018 | Atlanta | **819** | 1 | **250** | 2 | H275Y | H275Y | Unknown | Unknown | Unknown | Panama | 2018/08/23 |
| 16 | A(H1N1)pdm09 | A/India/1561/2019 | Atlanta | **1376** | 3 | **746** | 3 | H275Y, G147R/G mix | Not available | Unknown | Unknown | Unknown | India | 2019/02/19 |
| 17 | A(H1N1)pdm09 | A/Phuket/74/2019 | Atlanta | **56** | 2 | **32** | 3 | H275Y/H mix | Not available | Unknown | Unknown | Unknown | Thailand | 2019/02/12 |
| 18 | A(H1N1)pdm09 | A/El Salvador/622/2018 | Atlanta | **11** | **11** | 1 | 2 | V116A | Not available | Unknown | Unknown | Unknown | El Salvador | 2018/08/09 |
| 19 | A(H1N1)pdm09 | A/Samara/RII-13/2019 | Atlanta | 1 | **13** | **15** | 9* | Q136K/Q mix, D151E/D mix | Not available | Unknown | Unknown | Unknown | Russia | 2019/02/07 |
| 20 | A(H1N1)pdm09 | A/Santiago/49675/2019 | Atlanta | 2 | **332** | **196** | **56** | Q136R | Not available | Unknown | Unknown | Unknown | Chile | 2019/04/06 |
| 21 | A(H1N1)pdm09 | A/New York/16/2020 | Atlanta | **22** | 4 | 3 | 3 | I223K | I223K | Unknown | Unknown | Unknown | United States | 2020/01/24 |
| 22 | A(H1N1)pdm09 | A/Argentina/264/2018 | Atlanta | **22** | 5 | 3 | 3 | I223K | I223K | Unknown | Unknown | Unknown | Argentina | 2018/07/24 |
| 23 | A(H1N1)pdm09 | A/Santiago/108999/2018 | Atlanta | **11** | 2 | 1 | 1 | I223L | I223L | Unknown | Unknown | Unknown | Chile | 2018/12/21 |
| 24 | A(H1N1)pdm09 | A/Iowa/73/2018 | Atlanta | **12** | 2 | 2 | 1 | I223M | I223M | Unknown | Unknown | Unknown | United States | 2018/12/11 |
| 25 | A(H1N1)pdm09 | A/Ontario/RV1604/2019 | Atlanta | **28** | 8* | **11** | 5 | I223R | Not available | Unknown | Unknown | Unknown | Canada | 2019/03/20 |
| 26 | A(H1N1)pdm09 | A/West Virginia/09/2020 | Atlanta | **191** | 3 | **13** | 4 | N295S | N295S | Unknown | Unknown | Unknown | United States | 2020/02/26 |
| 27 | A(H1N1)pdm09 | A/Liaoning-Laobian/SWL1171/2020 | Beijing | **406** | 1 | n/t^e^ | n/t | H275Y | Not available | Hospital | Unknown | No | China | 2020/02/06 |
| 28 | A(H1N1)pdm09 | A/Jiangxi-Yuehu/SWL1185/2019 | Beijing | **436** | 1 | n/t | n/t | H275Y | Not available | Hospital | Unknown | No | China | 2019/02/12 |
| 29 | A(H1N1)pdm09 | A/Jiangxi-Zhanggong/SWL169/2019 | Beijing | **438** | 1 | n/t | n/t | H275Y | Not available | Hospital | Unknown | No | China | 2019/01/21 |
| 30 | A(H1N1)pdm09 | A/Zhejiang-Nanhu/SWL140/2019 | Beijing | **520** | 1 | n/t | n/t | H275Y | Not available | Hospital | Unknown | No | China | 2019/01/14 |
| 31 | A(H1N1)pdm09 | A/Shandong-Laoshan/SWL310/2019 | Beijing | **836** | 2 | n/t | n/t | H275Y | Not available | Hospital | Unknown | No | China | 2019/01/14 |
| 32 | A(H1N1)pdm09 | A/Shandong-Dongchangfu/SWL17/2019 | Beijing | **605** | 2 | n/t | n/t | H275Y | Not available | Hospital | Unknown | No | China | 2019/01/01 |
| 33 | A(H1N1)pdm09 | A/Neimenggu-Dongsheng/SWL1623/2018 | Beijing | **747** | 1 | n/t | n/t | H275Y | Not available | Hospital | Unknown | No | China | 2018/12/24 |
| 34 | A(H1N1)pdm09 | A/Henan-Weidou/SWL1588/2018 | Beijing | **810** | 2 | n/t | n/t | H275Y | Not available | Hospital | Unknown | No | China | 2018/12/22 |
| 35 | A(H1N1)pdm09 | A/Neimenggu-Donghe/SWL1610/2018 | Beijing | **853** | 1 | n/t | n/t | H275Y | Not available | Hospital | Unknown | No | China | 2018/12/17 |
| 36 | A(H1N1)pdm09 | A/Zhejiang-Haishu/SWL12011/2018 | Beijing | **604** | 1 | n/t | n/t | H275Y | Not available | Hospital | Unknown | No | China | 2018/12/16 |
| 37 | A(H1N1)pdm09 | A/Neimenggu-Dongsheng/SWL1584/2018 | Beijing | **852** | 1 | n/t | n/t | H275Y | Not available | Hospital | Unknown | No | China | 2018/12/10 |
| 38 | A(H1N1)pdm09 | A/Fujian-Gulou/SWL1994/2018 | Beijing | **1476** | 1 | n/t | n/t | H275Y | Not available | Hospital | Unknown | No | China | 2018/12/10 |
| 39 | A(H1N1)pdm09 | A/Neimenggu-Donghe/SWL1554/2018 | Beijing | **320** | 1 | n/t | n/t | H275Y | Not available | Hospital | Unknown | No | China | 2018/11/26 |
| 40 | A(H1N1)pdm09 | A/Fujian-Pinghe/SWL34/2018 | Beijing | **1337** | 1 | n/t | n/t | H275Y | Not available | Hospital | Unknown | No | China | 2018/11/22 |
| 41 | A(H1N1)pdm09 | A/Yunnan-Simao/SWL1937/2018 | Beijing | **1895** | 1 | n/t | n/t | H275Y | Not available | Hospital | Unknown | No | China | 2018/11/19 |
| 42 | A(H1N1)pdm09 | A/Xinjiang-Tianshan/SWL11167/2018 | Beijing | **1059** | 1 | n/t | n/t | H275Y | Not available | Hospital | Unknown | No | China | 2018/11/17 |
| 43 | A(H1N1)pdm09 | A/Neimenggu-Donghe/SWL1514/2018 | Beijing | **932** | 1 | n/t | n/t | H275Y | Not available | Hospital | Unknown | No | China | 2018/11/12 |
| 44 | A(H1N1)pdm09 | A/Xinjiang-Midong/SWL1565/2018 | Beijing | **1474** | 1 | n/t | n/t | H275Y | Not available | Hospital | Unknown | No | China | 2018/11/03 |
| 45 | A(H1N1)pdm09 | A/Xinjiang-Midong/SWL1561/2018 | Beijing | **1019** | 1 | n/t | n/t | H275Y | Not available | Hospital | Unknown | No | China | 2018/11/01 |
| 46 | A(H1N1)pdm09 | A/Zinjiang-Midong/SWL1558/2018 | Beijing | **1017** | 1 | n/t | n/t | H275Y | Not available | Hospital | Unknown | No | China | 2018/10/31 |
| 47 | A(H1N1)pdm09 | A/Jiangsu-Tinghu/SWL11150/2018 | Beijing | **687** | 1 | n/t | n/t | H275Y | Not available | Hospital | Unknown | No | China | 2018/07/16 |
| 48 | A(H1N1)pdm09 | A/Guangdong-Duanzhou/SWL1511/2018 | Beijing | **780** | 1 | n/t | n/t | H275Y | Not available | Hospital | Unknown | No | China | 2018/06/12 |
| 49 | A(H1N1)pdm09 | A/Jilin-Nanguan/SWL1106/2020 | Beijing | **114** | 4 | n/t | n/t | H275Y/H mix | Not available | Hospital | Unknown | No | China | 2020/02/11 |
| 50 | A(H1N1)pdm09 | A/Guangdong-Huadou/SWL3163/2018 | Beijing | **24** | 1 | n/t | n/t | H275Y/H mix | Not available | Hospital | Unknown | No | China | 2018/12/05 |
| 51 | A(H1N1)pdm09 | A/Hubei-Echeng/SWL11234/2019 | Beijing | 1 | **29** | n/t | n/t | Q136K | Not available | Hospital | Unknown | No | China | 2019/01/04 |
| 52 | A(H1N1)pdm09 | A/Lund/4/2020 | London | **388** | n/t | n/t | n/t | H275Y | Yes, H275X | Hospital | Yes, oseltamivir | Unknown | Sweden | 2020/02/17 |
| 53 | A(H1N1)pdm09 | A/Muscat/6997/2019 | London | **370** | n/t | n/t | n/t | H275Y | H275Y | Unknown | Unknown | Unknown | Oman | 2019/10/29 |
| 54 | A(H1N1)pdm09 | A/Trnava/535/2019 | London | **286** | 1 | n/t | n/t | H275Y | Not available | Hospital | Unknown | Unknown | Slovakia | 2019/04/10 |
| 55 | A(H1N1)pdm09 | A/Antalya/5/2018 | London | **734** | 3 | n/t | n/t | H275Y | Not available | Hospital | Unknown | Unknown | Turkey | 2018/12/18 |
| 56 | A(H1N1)pdm09 | A/Sur/6980/2019 | London | **232** | n/t | n/t | n/t | H275Y/H mix | H275Y/H mix | Unknown | Unknown | Unknown | Oman | 2019/10/27 |
| 57 | A(H1N1)pdm09 | A/Salalah/6780/2019 | London | **30** | n/t | n/t | n/t | H275Y/H mix | H275Y/H mix | Unknown | Unknown | Unknown | Oman | 2019/10/23 |
| 58 | A(H1N1)pdm09 | A/Denmark/3311/2019 | London | n/t | **280** | n/t | n/t | Q136K | Not available | Unknown | Unknown | Unknown | Denmark | 2019/11/16 |
| 59 | A(H1N1)pdm09 | A/Denmark/3295/2019 | London | n/t | **143** | n/t | n/t | Q136K | Not available | Unknown | Unknown | Unknown | Denmark | 2019/11/15 |
| 60 | A(H1N1)pdm09 | A/Mauritius/I-545/2018 | London | 0.5 | **254** | n/t | n/t | Q136K | Not available | Hospital | Unknown | Unknown | Mauritius | 2018/07/08 |
| 61 | A(H1N1)pdm09 | A/Denmark/2697/2019 | London | 1.5 | **38** | n/t | n/t | Q136K/Q mix, D151E/D mix | Not available | Unknown | Unknown | Unknown | Denmark | 2019/03/14 |
| 62 | A(H1N1)pdm09 | A/Hong Kong/133/2019 | London | **19** | 4 | n/t | n/t | I223K | Not available | Unknown | Unknown | Unknown | Hong Kong | 2018/12/31 |
| 63 | A(H1N1)pdm09 | A/Cyprus/919/2019 | London | **116** | 4 | n/t | n/t | N295S | N295S | Hospital | Yes | Unknown | Cyprus | 2019/02/13 |
| 64 | A(H1N1)pdm09 | A/Perth/24/2020 | Melbourne | **999** | 1 | **220** | 4 | H275Y | H275Y | Hospital | Unknown | Unknown | Australia | 2020/02/05 |
| 65 | A(H1N1)pdm09 | A/Perth/22/2020 | Melbourne | **1082** | 1 | **236** | 4 | H275Y | H275Y | Hospital | Unknown | Unknown | Australia | 2020/02/02 |
| 66 | A(H1N1)pdm09 | A/Townsville/32/2019 | Melbourne | **849** | 1 | **137** | 3 | H275Y | H275Y | Hospital | Unknown | Unknown | Australia | 2019/04/24 |
| 67 | A(H1N1)pdm09 | A/Victoria/2183/2018 | Melbourne | **1340** | 2 | **251** | 3 | H275Y | H275Y | Hospital | Unknown | Unknown | Australia | 2018/12/17 |
| 68 | A(H1N1)pdm09 | A/Sydney/29/2020 | Melbourne | 2 | **26** | 9 | 6 | E119K | None^d^ | Hospital | Unknown | Unknown | Australia | 2020/03/21 |
| 69 | A(H1N1)pdm09 | A/Victoria/2618/2019 | Melbourne | 1 | **321** | **24** | **58** | E119G | None | Hospital | Unknown | Unknown | Australia | 2019/06/12 |
| 70 | A(H1N1)pdm09 | A/Sydney/30/2020 | Melbourne | 1 | **22** | **19** | 5* | Q136K | None | Hospital | Unknown | Unknown | Australia | 2020/03/23 |
| 71 | A(H1N1)pdm09 | A/Philippines/13/2019 | Melbourne | 1 | **26** | **29** | 9* | Q136R | Not available | Hospital | Unknown | Unknown | Philippines | 2019/01/03 |
| 72 | A(H1N1)pdm09 | A/Cheboksary/125/2020 | Melbourne | **52** | **21** | n/t | n/t | R152K | Not available | Hospital | Unknown | Unknown | Russia | 2020/01/29 |
| 73 | A(H1N1)pdm09 | A/South Africa/1520/2020 | Melbourne | **12** | 3 | 2 | 1 | D199G | None | Unknown | Unknown | Unknown | South Africa | 2020/02/10 |
| 74 | A(H1N1)pdm09 | A/Malaysia/RP4517/2018 | Melbourne | **96** | 3 | 2* | 3 | N295S | Not available | Unknown | Unknown | Unknown | Malaysia | 2018/12/12 |
| 75 | A(H1N1)pdm09 | A/Brisbane/13/2020 | Melbourne | 1 | **81** | **83** | 7* | E119K, Q136K | None | Hospital | Unknown | Unknown | Australia | 2020/02/25 |
| 76 | A(H1N1)pdm09 | A/Shiga/13/2020 | Tokyo | **1421** | 1 | **272** | 1 | H275Y | H275Y | Community | Yes, peramivir | No | Japan | 2020/02/10 |
| 77 | A(H1N1)pdm09 | A/Niigata/145/2020 | Tokyo | **2194** | 2 | **629** | 3 | H275Y | H275Y/H mix | Community | Yes, peramivir, laninamivir | No | Japan | 2020/02/07 |
| 78 | A(H1N1)pdm09 | A/Niigata/114/2020 | Tokyo | **1196** | 1 | **254** | 3 | H275Y | H275Y/H mix | Community | Yes, oseltamivir | No | Japan | 2020/01/23 |
| 79 | A(H1N1)pdm09 | A/Oita/1/2020 | Tokyo | **1308** | 1 | **296** | 1 | H275Y | H275Y | Community | Yes, peramivir | No | Japan | 2020/01/20 |
| 80 | A(H1N1)pdm09 | A/Fukui/5/2020 | Tokyo | **1328** | 1 | **247** | 2 | H275Y | H275Y | Community | No | No | Japan | 2020/01/19 |
| 81 | A(H1N1)pdm09 | A/Mie/2/2020 | Tokyo | **1256** | 1 | **229** | 3 | H275Y | H275Y | Hospital | Yes, oseltamivir | No | Japan | 2020/01/17 |
| 82 | A(H1N1)pdm09 | A/Aichi/369/2019 | Tokyo | **1616** | 1 | **350** | 2 | H275Y | H275Y | Community | No | No | Japan | 2019/12/30 |
| 83 | A(H1N1)pdm09 | A/Kanagawa/271/2019 | Tokyo | **1524** | 1 | **262** | 2 | H275Y | H275Y | Community | No | No | Japan | 2019/12/20 |
| 84 | A(H1N1)pdm09 | A/Toyama/57/2019 | Tokyo | **1299** | 0.5 | **367** | 1 | H275Y | H275Y | Community | No | No | Japan | 2019/12/18 |
| 85 | A(H1N1)pdm09 | A/Shiga/30/2019 | Tokyo | **1090** | 1 | **211** | 2 | H275Y | H275Y | Community | No | No | Japan | 2019/12/18 |
| 86 | A(H1N1)pdm09 | A/Kanagawa/AC1926/2019 | Tokyo | **956** | 1 | **225** | 4 | H275Y | H275Y | Community | No | No | Japan | 2019/12/16 |
| 87 | A(H1N1)pdm09 | A/Ibaraki/129/2019 | Tokyo | **1212** | 2 | **253** | 3 | H275Y | H275Y | Community | No | No | Japan | 2019/12/16 |
| 88 | A(H1N1)pdm09 | A/Kawasaki/280/2019 | Tokyo | **1228** | 1 | **199** | 3 | H275Y | H275Y | Hospital | Yes, oseltamivir | Unknown | Japan | 2019/12/16 |
| 89 | A(H1N1)pdm09 | A/Sakai/82/2019 | Tokyo | **1437** | 1 | **307** | 2 | H275Y | H275Y | Community | Yes, oseltamivir | Unknown | Japan | 2019/12/14 |
| 90 | A(H1N1)pdm09 | A/Kawasaki/281/2019 | Tokyo | **1405** | 1 | **245** | 3 | H275Y | H275Y | Hospital | Yes, oseltamivir | Unknown | Japan | 2019/12/14 |
| 91 | A(H1N1)pdm09 | A/Aomori/60/2019 | Tokyo | **1100** | 1 | **236** | 2 | H275Y | H275Y/H mix | Community | No | No | Japan | 2019/12/12 |
| 92 | A(H1N1)pdm09 | A/Iwate/65/2019 | Tokyo | **1210** | 1 | **177** | 2 | H275Y | H275Y | Community | No | No | Japan | 2019/12/10 |
| 93 | A(H1N1)pdm09 | A/Sakai/71/2019 | Tokyo | **1298** | 1 | **265** | 2 | H275Y | H275Y | Community | Yes, peramivir | No | Japan | 2019/12/04 |
| 94 | A(H1N1)pdm09 | A/Kawasaki/214/2019 | Tokyo | **1614** | 2 | **414** | 3 | H275Y | H275Y | Community | No | No | Japan | 2019/12/02 |
| 95 | A(H1N1)pdm09 | A/Hiroshima-C/52/2019 | Tokyo | **1312** | 1 | **294** | 1 | H275Y | H275Y | Community | Unknown | No | Japan | 2019/11/26 |
| 96 | A(H1N1)pdm09 | A/Kanagawa/202/2019 | Tokyo | **1143** | 1 | **327** | 2 | H275Y | H275Y | Community | No | No | Japan | 2019/11/21 |
| 97 | A(H1N1)pdm09 | A/Tokyo/19390/2019 | Tokyo | **1194** | 2 | **269** | 2 | H275Y | Not available | Community | No | No | Japan | 2019/11/19 |
| 98 | A(H1N1)pdm09 | A/Tokyo/19387/2019 | Tokyo | **1263** | 2 | **239** | 3 | H275Y | Not available | Community | No | No | Japan | 2019/11/18 |
| 99 | A(H1N1)pdm09 | A/Yokohama/269/2019 | Tokyo | **1234** | 2 | **285** | 3 | H275Y | H275Y | Community | No | No | Japan | 2019/11/17 |
| 100 | A(H1N1)pdm09 | A/Iwate/56/2020 | Tokyo | **1366** | 2 | **332** | 4 | H275Y | H275Y | Community | No | No | Japan | 2019/11/16 |
| 101 | A(H1N1)pdm09 | A/IWATE/56/2019 | Tokyo | **1366** | 2 | 332 | 4 | H275Y | H275Y | Community | No | No | Tokyo | 2019/11/16 |
| 102 | A(H1N1)pdm09 | A/Iwate/55/2019 | Tokyo | **1639** | 1 | **265** | 4 | H275Y | H275Y | Community | No | No | Japan | 2019/11/15 |
| 103 | A(H1N1)pdm09 | A/Tokyo/19370/2019 | Tokyo | **1235** | 1 | **255** | 1 | H275Y | Not available | Community | No | No | Japan | 2019/11/11 |
| 104 | A(H1N1)pdm09 | A/Kanagawa/193/2019 | Tokyo | **1134** | 1 | **226** | 1 | H275Y | H275Y | Community | No | No | Japan | 2019/11/08 |
| 105 | A(H1N1)pdm09 | A/Kanagawa/ZC1905/2019 | Tokyo | **1193** | 1 | **305** | 1 | H275Y | H275Y/H mix | Community | Yes, oseltamivir | No | Japan | 2019/10/23 |
| 106 | A(H1N1)pdm09 | A/Ehime/95/2019 | Tokyo | **1266** | 1 | **206** | 2 | H275Y | Not available | Community | Yes, oseltamivir | No | Japan | 2019/10/08 |
| 107 | A(H1N1)pdm09 | A/Ehime/96/2019 | Tokyo | **1362** | 1 | **187** | 2 | H275Y | H275Y | Community | Yes, laninamivir | No | Japan | 2019/10/06 |
| 108 | A(H1N1)pdm09 | A/Ehime/94/2019 | Tokyo | **1187** | 1 | **278** | 1 | H275Y | H275Y | Community | No | No | Japan | 2019/10/05 |
| 109 | A(H1N1)pdm09 | A/Ehime/93/2019 | Tokyo | **1053** | 1 | **252** | 2 | H275Y | H275Y | Community | No | No | Japan | 2019/09/17 |
| 110 | A(H1N1)pdm09 | A/Aomori/46/2019 | Tokyo | **1097** | 1 | **264** | 3 | H275Y | H275Y | Community | No | No | Japan | 2019/09/14 |
| 111 | A(H1N1)pdm09 | A/Okinawa/97/2019 | Tokyo | **1326** | 2 | **295** | 2 | H275Y | H275Y | Community | Yes, oseltamivir | Unknown | Japan | 2019/09/13 |
| 112 | A(H1N1)pdm09 | A/Ehime/85/2019 | Tokyo | **1132** | 2 | **286** | 3 | H275Y | H275Y/H mix | Hospital | Yes, oseltamivir, laninamivir | Yes | Japan | 2019/08/28 |
| 113 | A(H1N1)pdm09 | A/Ehime/86/2019 | Tokyo | **1209** | 2 | **294** | 3 | H275Y | H275Y | Hospital | Yes, oseltamivir, laninamivir | Yes | Japan | 2019/08/28 |
| 114 | A(H1N1)pdm09 | A/Ehime/87/2019 | Tokyo | **1473** | 2 | **312** | 4 | H275Y | H275Y | Hospital | Yes, oseltamivir, laninamivir | Yes | Japan | 2019/08/28 |
| 115 | A(H1N1)pdm09 | A/Ibaraki/93/2019 | Tokyo | **1320** | 2 | **246** | 3 | H275Y | H275Y | Community | Yes, oseltamivir | Unknown | Japan | 2019/07/18 |
| 116 | A(H1N1)pdm09 | A/Mie/15/2019 | Tokyo | **1740** | 2 | **387** | 4 | H275Y | H275Y | Hospital | Yes, oseltamivir | Unknown | Japan | 2019/03/15 |
| 117 | A(H1N1)pdm09 | A/Yokohama/141/2019 | Tokyo | **1013** | 2 | **279** | 3 | H275Y | H275Y | Hospital | Yes, oseltamivir | Yes | Japan | 2019/03/08 |
| 118 | A(H1N1)pdm09 | A/Yokohama/142/2019 | Tokyo | **1240** | 2 | **344** | 5 | H275Y | H275Y | Hospital | Yes, oseltamivir | Yes | Japan | 2019/03/08 |
| 119 | A(H1N1)pdm09 | A/Kanagawa/138/2019 | Tokyo | **1395** | 1 | **335** | 4 | H275Y | H275Y | Hospital | Yes, oseltamivir | Unknown | Japan | 2019/02/21 |
| 120 | A(H1N1)pdm09 | A/Kagawa/19135/2019 | Tokyo | **1832** | 1 | **416** | 4 | H275Y | H275Y | Hospital | Yes, oseltamivir | Unknown | Japan | 2019/02/20 |
| 121 | A(H1N1)pdm09 | A/Shizuoka-C/7/2019 | Tokyo | **1035** | 2 | **312** | 4 | H275Y | H275Y | Community | No | No | Japan | 2019/02/12 |
| 122 | A(H1N1)pdm09 | A/Saitama-C/13/2019 | Tokyo | **1110** | 2 | **265** | 3 | H275Y | H275Y | Hospital | Yes, peramivir | Unknown | Japan | 2019/02/01 |
| 123 | A(H1N1)pdm09 | A/Aichi/169/2019 | Tokyo | **593** | 1 | **131** | 1 | H275Y | H275Y | Community | No | No | Japan | 2019/02/01 |
| 124 | A(H1N1)pdm09 | A/Nagano/2126/2019 | Tokyo | **1420** | 2 | **439** | 5 | H275Y | H275Y | Hospital | Yes, oseltamivir | Unknown | Japan | 2019/01/31 |
| 125 | A(H1N1)pdm09 | A/Fukui/13/2019 | Tokyo | **1185** | 2 | **464** | 3 | H275Y | H275Y | Community | Yes, oseltamivir | No | Japan | 2019/01/28 |
| 126 | A(H1N1)pdm09 | A/Aichi/106/2019 | Tokyo | **1518** | 1 | **444** | 1 | H275Y | H275Y | Community | No | No | Japan | 2019/01/28 |
| 127 | A(H1N1)pdm09 | A/Kawasaki/70/2019 | Tokyo | **1271** | 1 | **314** | 3 | H275Y | H275Y | Community | Yes, oseltamivir, baloxavir | No | Japan | 2019/01/25 |
| 128 | A(H1N1)pdm09 | A/Wakayama-C/34/2019 | Tokyo | **1622** | 3 | **419** | 5 | H275Y | H275Y | Hospital | Yes, peramivir | Unknown | Japan | 2019/01/22 |
| 129 | A(H1N1)pdm09 | A/Shiga/25/2018 | Tokyo | **1324** | 2 | **395** | 4 | H275Y | H275Y | Community | Yes, oseltamivir | No | Japan | 2018/12/28 |
| 130 | A(H1N1)pdm09 | A/Nagano/2649/2018 | Tokyo | **1337** | 1 | **348** | 2 | H275Y | H275Y | Community | Yes, oseltamivir | No | Japan | 2018/11/26 |
| 131 | A(H1N1)pdm09 | A/Aichi/200/2018 | Tokyo | **1478** | 1 | **331** | 2 | H275Y | H275Y | Community | No | No | Japan | 2018/08/22 |
| 132 | A(H1N1)pdm09 | A/Ibaraki/139/2019 | Tokyo | **976** | 2 | **197** | 3 | H275Y/H mix | None | Community | No | No | Japan | 2019/11/30 |
| 133 | A(H1N1)pdm09 | A/Toyama/21/2019 | Tokyo | 1 | **42** | **16** | 8* | Q136K/Q mix, D151N/D mix | None | Community | No | No | Japan | 2019/01/21 |
|  |  |  |  |  |  |  |  |  |  |  |  |  |  |  |
| 1 | A(H3N2) | A/Yokohama/95/2018 | Tokyo | 6 | **12** | 2 | 4 | D151N/D mix | None | Community | Unknown | No | Japan | 2018/09/27 |
| 2 | A(H3N2) | A/Latvia/03-0738053/2019 | London | 4.5 | **21** | n/t | n/t | D151N/D mix, V165I | Not available | Sentinel outpatient | No | Unknown | Latvia | 2019/02/26 |
| 3 | A(H3N2) | A/Finland/108/2019 | London | 1 | **17** | n/t | n/t | M241V/M mix | Not available | Unknown | Yes | Unknown | Finland | 2019/04/25 |
| 4 | A(H3N2) | A/Wakayama-C/38/2019 | Tokyo | **15** | 5 | 1.5 | 1 | N245Y(-gly) | N245Y(-gly) | Hospital | Yes, peramivir | Unknown | Japan | 2019/03/27 |
| 5 | A(H3N2) | A/Wakayama/17/2020 | Tokyo | **11.5** | 4 | 1 | 2 | S331R | S331R | Community | No | No | Japan | 2020/01/12 |

^a^ RI and HRI fold-change values are displayed underlined and in bold typeface.

^b^ Amino acid position numbering is A-subtype specific.

^c^ Clinical specimen not available for sequencing.

^d^ None: the virus contained no amino acid substitutions when compared to viruses with a normal inhibition (NI) phenotype.

^e^ n/t: not tested.

* Fold-changes in the NI range whereas fold-changes in the WHO reference table (<https://cdn.who.int/media/docs/default-source/influenza/nai-reduced-susceptibility-marker-table-who_table-1.pdf>) associated with the amino acid change(s) indicated range from NI up to HRI for particular drug and amino acid change, or other virus(es) in this supplementary table with the same amino acid change(s) have RI phenotype for the indicated antiviral drug. Values marked are in the range upper whisker fence in the box and whisker plot and threshold for RI phenotype.

**Table S4.** Influenza B/Victoria-lineage and B/Yamagata-lineage virus isolates showing RI or HRI by one or more NAIs (n = 55).

| # | Type/lineage | Strain designation | WHO CC | IC_50_ fold-change as compared to reference median IC_50_^a^ | | | | Substitution in virus isolate^b^ | Substitution in original specimen^b^ | Patient  setting | Antiviral  treatment | Immuno-compromised? | Country of specimen collection | Date of collection (y/m/d) |
| --- | --- | --- | --- | --- | --- | --- | --- | --- | --- | --- | --- | --- | --- | --- |
|  |  |  |  | Oseltamivir | Zanamivir | Peramivir | Laninamivir |  |  |  |  |  |  |  |
| 1 | B/Vic | B/California/31/2020 | Atlanta | 2* | 4.5* | 3* | 4 | D197N | D197N | Unknown | Unknown | Unknown | United States | 2020/03/04 |
| 2 | B/Vic | B/Delaware/11/2019 | Atlanta | 4* | **8** | 3* | 4 | D197N | D197N | Unknown | Unknown | Unknown | United States | 2019/10/18 |
| 3 | B/Vic | B/Guatemala/5522/2019 | Atlanta | 4* | **5** | 3* | 3 | D197N | D197N | Unknown | Unknown | Unknown | Guatemala | 2019/05/29 |
| 4 | B/Vic | B/Texas/26/2019 | Atlanta | **7** | 2 | **10** | 2 | I221T | I221T | Unknown | Unknown | Unknown | United States | 2019/05/22 |
| 5 | B/Vic | B/New Jersey/02/2020 | Atlanta | **19** | **22** | **58** | 3 | G243S | G243S | Unknown | Unknown | Unknown | United States | 2020/01/19 |
| 6 | B/Vic | B/Maryland/15/2019 | Atlanta | 1 | 1 | **61** | 1 | H273Y | H273Y | Unknown | Unknown | Unknown | United States | 2019/04/10 |
| 7 | B/Vic | B/Guangxi-Xinbin/1494/2019 | Beijing | 4* | **6** | n/t^d^ | n/t | D197N | Not available^c^ | Hospital | Unknown | No | China | 2019/05/27 |
| 8 | B/Vic | B/Guangxi-Xinbin/1668/2019 | Beijing | **23** | **257** | n/t | n/t | G243D | Not available | Hospital | Unknown | Unknown | China | 2019/07/23 |
| 9 | B/Vic | B/Norway/3241/2018 | London | 4* | **6** | n/t | n/t | D197N | D197N | Hospital | Unknown | Unknown | Norway | 2018/08/21 |
| 10 | B/Vic | B/Estonia/125782/2020 | London | 3 | **5** | n/t | n/t | I348V | Not available | Hospital | Unknown | Unknown | Estonia | 2020/01/13 |
| 11 | B/Vic | B/Malaysia/RP2136/2019 | Melbourne | 5 | **55** | **829** | 3 | H101L | Not available | Unknown | Unknown | Unknown | Malaysia | 2019/04/07 |
| 12 | B/Vic | B/Brunei/16/2019 | Melbourne | **6** | **8** | **102** | 3* | E105K | Not available | Unknown | Unknown | Unknown | Brunei | 2019/09/11 |
| 13 | B/Vic | B/Canberra/65/2019 | Melbourne | **10** | **72** | **684** | **6** | E105K | None^e^ | Hospital | Unknown | Unknown | Australia | 2019/09/09 |
| 14 | B/Vic | B/Malaysia/UM004/2019 | Melbourne | 1 | 3* | **6** | 2* | E105K/E mix | Not available | Hospital | Unknown | Unknown | Malaysia | 2019/07/07 |
| 15 | B/Vic | B/New Caledonia/2/2019 | Melbourne | 1 | 2* | **17** | 2* | E105K | None | Hospital | Unknown | Unknown | New Caledonia | 2019/05/27 |
| 16 | B/Vic | B/Malaysia/RP1208/2019 | Melbourne | 2* | **67** | **607** | **7** | E105K/E mix | Not available | Unknown | Unknown | Unknown | Malaysia | 2019/02/22 |
| 17 | B/Vic | B/Malaysia/RP1204/2019 | Melbourne | 4* | **78** | **1517** | **13** | E105K | Not available | Unknown | Unknown | Unknown | Malaysia | 2019/02/19 |
| 18 | B/Vic | B/Malaysia/RP0750/2018 | Melbourne | 1 | 2* | **5** | 2* | E105K, K382R | Not available | Unknown | Unknown | Unknown | Malaysia | 2018/11/13 |
| 19 | B/Vic | B/Philippines/6/2018 | Melbourne | 4* | **7** | **161** | 3* | E105K | E105K/E mix | Hospital | No | Unknown | Philippines | 2018/11/05 |
| 20 | B/Vic | B/Prachuapkhirikhan/247/2019 | Melbourne | **6** | **5** | **18** | 4 | G108E | Not available | Hospital | Yes, oseltamivir | Unknown | Thailand | 2019/05/20 |
| 21 | B/Vic | B/South Australia/127/2019 | Melbourne | 4 | 2 | **32** | 1 | H134Y/H mix | None | Unknown | Unknown | Unknown | Australia | 2019/10/19 |
| 22 | B/Vic | B/Fiji/68/2019 | Melbourne | 3 | 3 | **32** | 2 | H134Y/H mix | None | Unknown | Unknown | Unknown | Fiji | 2019/05/13 |
| 23 | B/Vic | B/Victoria/937/2019 | Melbourne | 0.1 | 2 | **6** | 1 | Q138K/Q mix | None | Hospital | Unknown | Unknown | Australia | 2019/06/19 |
| 24 | B/Vic | B/Townsville/9/2019 | Melbourne | **7** | **12** | **99** | 4.5* | G140R | None | Hospital | Unknown | Unknown | Australia | 2019/09/10 |
| 25 | B/Vic | B/Brisbane/6/2019 | Melbourne | **6** | **41** | **259** | **6** | G140R | None | Hospital | Unknown | Unknown | Australia | 2019/05/17 |
| 26 | B/Vic | B/Canberra/29/2019 | Melbourne | 3* | 3* | **6** | 1 | G145R | Not available | Emergency | Unknown | Unknown | Australia | 2019/06/05 |
| 27 | B/Vic | B/Singapore/TT0473/2020 | Melbourne | 0.5 | 4 | **51** | 2 | T146K | None | Hospital | No | Unknown | Singapore | 2020/01/29 |
| 28 | B/Vic | B/Brisbane/27/2019 | Melbourne | 3 | 3 | **418** | 2 | T146P | None | Hospital | Unknown | Unknown | Australia | 2019/09/01 |
| 29 | B/Vic | B/Malaysia/RP2134/2019 | Melbourne | **29** | **87** | **5707** | **6** | T146P, N169S | Not available | Unknown | Unknown | Unknown | Malaysia | 2019/06/26 |
| 30 | B/Vic | B/Philippines/12/2018 | Melbourne | **17** | **9** | **5763** | 2 | T146K | None | Hospital | Unknown | Unknown | Philippines | 2018/11/05 |
| 31 | B/Vic | B/Philippines/5/2018 | Melbourne | **5** | 3 | **7** | 2 | I221T/I mix | None | Hospital | No | Unknown | Philippines | 2018/11/12 |
| 32 | B/Vic | B/Philippines/5/2020 | Melbourne | 4* | **15** | 3* | 2* | A245T | None | Hospital | No | Unknown | Philippines | 2020/01/21 |
| 33 | B/Vic | B/Malaysia/RP1210/2019 | Melbourne | 2 | 4 | **33** | 2 | S34L, K382R | Not available | Unknown | Unknown | Unknown | Malaysia | 2019/02/26 |
| 34 | B/Vic | B/Malaysia/RP2129/2019 | Melbourne | 1 | 2 | **52** | 1 | D432G | Not available | Unknown | Unknown | Unknown | Malaysia | 2019/06/13 |
| 35 | B/Vic | B/Victoria/35/2019 | Melbourne | 0.1 | 2 | **27** | 1 | H439R | None | Unknown | Unknown | Unknown | Australia | 2019/08/28 |
| 36 | B/Vic | B/Malaysia/RP2127/2019 | Melbourne | 0.4 | **15** | **200** | 3 | H439P | Not available | Unknown | Unknown | Unknown | Malaysia | 2019/06/13 |
| 37 | B/Vic | B/Brisbane/31/2019 | Melbourne | 4 | 3 | **18** | 2 | T460I | None | Emergency | Unknown | Unknown | Australia | 2019/09/09 |
| 38 | B/Vic | B/Brisbane/3/2019 | Melbourne | 2 | 2 | **23** | 2 | H101L, K358R | K358R | Community | Unknown | Unknown | Australia | 2019/03/15 |
| 39 | B/Vic | B/Brisbane/1004/2019 | Melbourne | 1 | 1 | **6** | 2* | G104R/G mix, E105K/E mix | None | Unknown | Unknown | Unknown | Australia | 2019/07/27 |
| 40 | B/Vic | B/Canberra/68/2019 | Melbourne | 1 | **8** | **13** | 3 | G104R, G145R | None | Emergency | No | Unknown | Australia | 2019/09/17 |
| 41 | B/Vic | B/Brisbane/32/2019 | Melbourne | 3* | **5** | 3* | 3* | E105K, I115del | None | Emergency | Unknown | Unknown | Australia | 2019/09/12 |
| 42 | B/Vic | B/Townsville/7/2019 | Melbourne | 1 | 1 | **18** | 1 | E105G, P139L | None | Emergency | Unknown | Unknown | Australia | 2019/07/28 |
| 43 | B/Vic | B/Malaysia/RP1211/2019 | Melbourne | **5** | **64** | **348** | **30** | E105K, P139T/P mix | Not available | Unknown | Unknown | Unknown | Malaysia | 2019/02/26 |
| 44 | B/Vic | B/Brisbane/12/2019 | Melbourne | 4 | 1 | **243** | 1 | H134Y, D432G | None | Hospital | Unknown | Unknown | Australia | 2019/07/08 |
| 45 | B/Vic | B/Townsville/12/2019 | Melbourne | 2 | **9** | **103** | 2 | G247D, I361V | G247D, I361V | Hospital | Unknown | Unknown | Australia | 2019/10/20 |
| 46 | B/Vic | B/Yamanashi/19249/2019 | Tokyo | 2 | 1 | **6** | 1 | H134Y/H mix | None | Community | Unknown | No | Japan | 2019/03/27 |
| 47 | B/Vic | B/Kanagawa/IC18167/2019 | Tokyo | 1 | 4 | **7** | 2 | Q138K/Q mix | None | Community | No | No | Japan | 2019/04/14 |
| 48 | B/Vic | B/Shiga/21/2019 | Tokyo | 2 | **9** | 5 | 3 | G145R | G145R | Community | No | No | Japan | 2019/04/24 |
|  |  |  |  |  |  |  |  |  |  |  |  |  |  |  |
| 1 | B/Yam | B/Wisconsin/23/2019 | Atlanta | **5** | 3* | 3* | 4 | D197N | D197N | Unknown | Unknown | Unknown | United States | 2019/04/30 |
| 2 | B/Yam | B/Argentina/13785/2018 | Atlanta | 1 | 1 | **7** | 2 | I221V | I221V | Unknown | Unknown | Unknown | Argentina | 2018/08/06 |
| 3 | B/Yam | B/Santiago/53060/2018 | Atlanta | 1 | **12** | 2 | 3 | S249N | Not available | Unknown | Unknown | Unknown | Chile | 2018/06/27 |
| 4 | B/Yam | B/New Hampshire/07/2019 | Atlanta | 0.3 | 1 | **22** | 1 | H273Y/H mix | H273Y/H mix | Unknown | Unknown | Unknown | United States | 2019/03/31 |
| 5 | B/Yam | B/Sydney/3/2019 | Melbourne | **7** | 3 | **9** | 1 | A200T | A200T | Hospital | Unknown | Unknown | Australia | 2019/01/22 |
| 6 | B/Yam | B/Sydney/1003/2018 | Melbourne | **8** | **5** | **13** | 4 | No mutation detected | Not sequenced | Community | Unknown | Unknown | Australia | 2018/05/22 |
| 7 | B/Yam | B/Aichi/9/2019 | Tokyo | **15** | 0.5 | **616** | 1 | H273Y | H273Y | Community | No | No | Japan | 2019/03/01 |

^a^ RI and HRI fold-change values are displayed underlined and in bold typeface.

^b^ Amino acid position numbering is B-type specific.

^c^ Clinical specimen not available for sequencing.

^d^ n/t: not tested.

^e^ None: the virus contained no amino acid substitutions when compared to viruses with a normal inhibition (NI) phenotype.

* Fold-changes in the NI range whereas fold-changes in the WHO reference table (<https://cdn.who.int/media/docs/default-source/influenza/nai-reduced-susceptibility-marker-table-who_table-1.pdf>) associated with the amino acid change(s) indicated range from NI up to HRI for particular drug and amino acid change, or other virus(es) in this supplementary table with the same amino acid change(s) have RI phenotype for the indicated antiviral drug. Values marked are in the range upper whisker fence in the box and whisker plot and threshold for RI phenotype.

**Table S5.** Influenza type A and B viruses carrying NA substitutions associated with RI/HRI on the basis of sequence analysis from GISAID (n = 131).^a^

| **#** | **Type/subtype or lineage** | **Strain designation** | **Submitting laboratory** | **NA substitution^b^** | **Passage details/history^c^** | **NA GISAID Acc. No.** | **Country of specimen collection** | **Period** |
| --- | --- | --- | --- | --- | --- | --- | --- | --- |
| **1** | A(H1N1)pdm09 | A/Japan/8141/2019 | U.S. Air Force School of Aerospace Medicine | H275Y | Original | EPI1517865 | Japan | 2018–2019 |
| **2** | A(H1N1)pdm09 | A/Kansas/8811/2019 | U.S. Air Force School of Aerospace Medicine | H275Y | Original | EPI1502737 | United States | 2018–2019 |
| **3** | A(H1N1)pdm09 | A/North Carolina/8184/2019 | U.S. Air Force School of Aerospace Medicine | H275Y | Original | EPI1517400 | United States | 2018–2019 |
| **4** | A(H1N1)pdm09 | A/Austria/1137060/2019 | Medical University of Vienna | H275Y | Cell | EPI1440547 | Austria | 2018–2019 |
| **5** | A(H1N1)pdm09 | A/Berlin/11/2019 | Robert Koch Institute | H275Y | Original | EPI1378210 | Germany | 2018–2019 |
| **6** | A(H1N1)pdm09 | A/Berlin/45/2019 | Robert Koch Institute | H275Y | Original | EPI1378218 | Germany | 2018–2019 |
| **7** | A(H1N1)pdm09 | A/Cardiff/6307/2019 | Public Health Wales Microbiology Cardiff | H275Y | Original | EPI1359921 | United Kingdom | 2018–2019 |
| **8** | A(H1N1)pdm09 | A/Centre/1027/2019 | Institut Pasteur | H275Y | Other | EPI1377914 | France | 2018–2019 |
| **9** | A(H1N1)pdm09 | A/England/190560518/2019 | Microbiology Reference Services, Colindale, Public Health England | H275Y | Original | EPI1796784 | United Kingdom | 2018–2019 |
| **10** | A(H1N1)pdm09 | A/England/190640778/2019 | Microbiology Reference Services, Colindale, Public Health England | H275Y | Original | EPI1796800 | United Kingdom | 2018–2019 |
| **11** | A(H1N1)pdm09 | A/England/190760513/2019 | Microbiology Reference Services, Colindale, Public Health England | H275Y | Original | EPI1796834 | United Kingdom | 2018–2019 |
| **12** | A(H1N1)pdm09 | A/England/79/2019 | Microbiology Reference Services, Colindale, Public Health England | H275Y | Original | EPI1418031 | United Kingdom | 2018–2019 |
| **13** | A(H1N1)pdm09 | A/England/81/2019 | Microbiology Reference Services, Colindale, Public Health England | H275Y | Original | EPI1417700 | United Kingdom | 2018–2019 |
| **14** | A(H1N1)pdm09 | A/England/82/2019 | Microbiology Reference Services, Colindale, Public Health England | H275Y | Original | EPI1417711 | United Kingdom | 2018–2019 |
| **15** | A(H1N1)pdm09 | A/England/84/2019 | Microbiology Reference Services, Colindale, Public Health England | H275Y | Original | EPI1491718 | United Kingdom | 2018–2019 |
| **16** | A(H1N1)pdm09 | A/Galicia/1492/2019 | Instituto de Salud Carlos III | H275Y | Original | EPI1520181 | Spain | 2018–2019 |
| **17** | A(H1N1)pdm09 | A/HK/2272/2018 | Centre for Health Protection | H275Y | Cell | EPI1324790 | Hong Kong (SAR) | 2018–2019 |
| **18** | A(H1N1)pdm09 | A/Korea/S0002/2019 | Korea Centers for Disease Control and Prevention | H275Y | Cell | EPI1602908 | Republic of Korea | 2018–2019 |
| **19** | A(H1N1)pdm09 | A/Korea/S0003/2019 | Korea Centers for Disease Control and Prevention | H275Y | Cell | EPI1602906 | Republic of Korea | 2018–2019 |
| **20** | A(H1N1)pdm09 | A/Nagasaki/18FS166/2018 | Niigata University (DPH) | H275Y | Cell | EPI1656973 | Japan | 2018–2019 |
| **21** | A(H1N1)pdm09 | A/Nagasaki/18FS166_2/2018 | Niigata University (DPH) | H275Y | Cell | EPI1656975 | Japan | 2018–2019 |
| **22** | A(H1N1)pdm09 | A/Netherlands/00445/2019 | Erasmus Medical Centre | H275Y | Original | EPI1377842 | Netherlands | 2018–2019 |
| **23** | A(H1N1)pdm09 | A/Okinawa/18T001/2018 | Niigata University (DPH) | H275Y | Cell | EPI1668783 | Japan | 2018–2019 |
| **24** | A(H1N1)pdm09 | A/Michigan/209/2019 | Centers for Disease Control and Prevention | H275Y | Original | EPI1461182 | United States | 2018–2019 |
| **25** | A(H1N1)pdm09 | A/Minnesota/28/2019 | Centers for Disease Control and Prevention | H275Y | Original | EPI1437677 | United States | 2018–2019 |
| **26** | A(H1N1)pdm09 | A/Minnesota/46/2019 | Centers for Disease Control and Prevention | H275Y | Original | EPI1546483 | United States | 2018–2019 |
| **27** | A(H1N1)pdm09 | A/Minnesota/47/2019 | Centers for Disease Control and Prevention | H275Y | Original | EPI1546397 | United States | 2018–2019 |
| **28** | A(H1N1)pdm09 | A/Nong Khai/288/2018 | Centers for Disease Control and Prevention | H275Y | Cell | EPI1363298 | Thailand | 2018–2019 |
| **29** | A(H1N1)pdm09 | A/Pennsylvania/658/2019 | Centers for Disease Control and Prevention | H275Y | Original | EPI1458367 | United States | 2018–2019 |
| **30** | A(H1N1)pdm09 | A/Texas/170/2019 | Centers for Disease Control and Prevention | H275Y | Original | EPI1563925 | United States | 2018–2019 |
| **31** | A(H1N1)pdm09 | A/England/2/2019 | Crick Worldwide Influenza Centre | H275Y | Cell | EPI1437206 | United Kingdom | 2018–2019 |
| **32** | A(H1N1)pdm09 | A/England/620/2018 | Crick Worldwide Influenza Centre | H275Y | Cell | EPI1437208 | United Kingdom | 2018–2019 |
| **33** | A(H1N1)pdm09 | A/Hunan-Hecheng/SWL1508/2019 | WHO Chinese National Influenza Center | H275Y | Cell | EPI1648990 | China | 2018–2019 |
| **34** | A(H1N1)pdm09 | A/Fujian-Sanyuan/SWL2680/2018 | WHO Chinese National Influenza Center | H275Y | Cell | EPI1339506 | China | 2018–2019 |
| **35** | A(H1N1)pdm09 | A/Fujian-Sanyuan/SWL2709/2018 | WHO Chinese National Influenza Center | H275Y | Cell | EPI1339470 | China | 2018–2019 |
| **36** | A(H1N1)pdm09 | A/Gansu-Xifeng/SWL1202/2019 | WHO Chinese National Influenza Center | H275Y | Cell | EPI1648993 | China | 2018–2019 |
| **37** | A(H1N1)pdm09 | A/Guangdong-Haizhu/SWL1682/2018 | WHO Chinese National Influenza Center | H275Y | Cell | EPI1339473 | China | 2018–2019 |
| **38** | A(H1N1)pdm09 | A/Jilin-Tiexi/SWL1281/2019 | WHO Chinese National Influenza Center | H275Y | Cell | EPI1648996 | China | 2018–2019 |
| **39** | A(H1N1)pdm09 | A/Japan/10014/2019 | U.S. Air Force School of Aerospace Medicine | H275Y | Original | EPI1657454 | Japan | 2019–2020 |
| **40** | A(H1N1)pdm09 | A//Romania/256678/2020 | Cantacuzino Institute | H275Y | Cell | EPI1745802 | Romania | 2019–2020 |
| **41** | A(H1N1)pdm09 | A/Alaska/11960/2020 | U.S. Air Force School of Aerospace Medicine | H275Y | Original | EPI1744489 | United States | 2019–2020 |
| **42** | A(H1N1)pdm09 | A/Alaska/12097/2020 | U.S. Air Force School of Aerospace Medicine | H275Y | Original | EPI1756723 | United States | 2019–2020 |
| **43** | A(H1N1)pdm09 | A/Ankara/14016-226/2020 | Gen Era Diagnostics Corporation | H275Y | Other | EPI1727443 | Turkey | 2019–2020 |
| **44** | A(H1N1)pdm09 | A/Budgam/AG_517/2020 | CNR Virus des Infections Respiratoires—France SUD | H275Y | Original | EPI1804036 | India | 2019–2020 |
| **45** | A(H1N1)pdm09 | A/California/12103/2020 | U.S. Air Force School of Aerospace Medicine | H275Y | Original | EPI1756762 | United States | 2019–2020 |
| **46** | A(H1N1)pdm09 | A/Georgia/10149/2019 | U.S. Air Force School of Aerospace Medicine | H275Y | Original | EPI1660047 | United States | 2019–2020 |
| **47** | A(H1N1)pdm09 | A/Georgia/10460/2019 | U.S. Air Force School of Aerospace Medicine | H275Y | Original | EPI1698951 | United States | 2019–2020 |
| **48** | A(H1N1)pdm09 | A/Georgia/10464/2019 | U.S. Air Force School of Aerospace Medicine | H275Y | Original | EPI1698979 | United States | 2019–2020 |
| **49** | A(H1N1)pdm09 | A/Georgia/10465/2019 | U.S. Air Force School of Aerospace Medicine | H275Y | Original | EPI1698987 | United States | 2019–2020 |
| **50** | A(H1N1)pdm09 | A/Georgia/10467/2019 | U.S. Air Force School of Aerospace Medicine | H275Y | Original | EPI1699003 | United States | 2019–2020 |
| **51** | A(H1N1)pdm09 | A/Georgia/10469/2019 | U.S. Air Force School of Aerospace Medicine | H275Y | Original | EPI1699018 | United States | 2019–2020 |
| **52** | A(H1N1)pdm09 | A/Georgia/10472/2019 | U.S. Air Force School of Aerospace Medicine | H275Y | Original | EPI1699041 | United States | 2019–2020 |
| **53** | A(H1N1)pdm09 | A/Georgia/9866/2019 | U.S. Air Force School of Aerospace Medicine | H275Y | Original | EPI1654092 | United States | 2019–2020 |
| **54** | A(H1N1)pdm09 | A/HK/3327/2019 | Centre for Health Protection | H275Y | Cell | EPI1604910 | Hong Kong (SAR) | 2019–2020 |
| **55** | A(H1N1)pdm09 | A/Japan/10158/2019 | U.S. Air Force School of Aerospace Medicine | H275Y | Original | EPI1660109 | Japan | 2019–2020 |
| **56** | A(H1N1)pdm09 | A/Japan/10159/2019 | U.S. Air Force School of Aerospace Medicine | H275Y | Original | EPI1663367 | Japan | 2019–2020 |
| **57** | A(H1N1)pdm09 | A/Japan/10160/2019 | U.S. Air Force School of Aerospace Medicine | H275Y | Original | EPI1660117 | Japan | 2019–2020 |
| **58** | A(H1N1)pdm09 | A/Japan/10162/2019 | U.S. Air Force School of Aerospace Medicine | H275Y | Original | EPI1660130 | Japan | 2019–2020 |
| **59** | A(H1N1)pdm09 | A/Mississippi/11165/2019 | U.S. Air Force School of Aerospace Medicine | H275Y | Original | EPI1709164 | United States | 2019–2020 |
| **60** | A(H1N1)pdm09 | A/Myanmar/19M027/2019 | Niigata University | H275Y | Cell | EPI1800250 | Myanmar | 2019–2020 |
| **61** | A(H1N1)pdm09 | A/Myanmar/19M052/2019 | Niigata University | H275Y | Cell | EPI1800243 | Myanmar | 2019–2020 |
| **62** | A(H1N1)pdm09 | A/New Brunswick/RV0091/2020 | Public Health Agency of Canada, National Microbiology Laboratory | H275Y | Other | EPI1659567 | Canada | 2019–2020 |
| **63** | A(H1N1)pdm09 | A/New Brunswick/RV0092/2020 | Public Health Agency of Canada, National Microbiology Laboratory | H275Y | Other | EPI1736626 | Canada | 2019–2020 |
| **64** | A(H1N1)pdm09 | A/Santiago/60457/2019 | Instituto de Salud Pública de Chile | H275Y | Original | EPI1568524 | Chile | 2019–2020 |
| **65** | A(H1N1)pdm09 | A/Texas/10344/2019 | U.S. Air Force School of Aerospace Medicine | H275Y | Original | EPI1668095 | United States | 2019–2020 |
| **66** | A(H1N1)pdm09 | A/Virginia/10555/2019 | U.S. Air Force School of Aerospace Medicine | H275Y | Original | EPI1699641 | United States | 2019–2020 |
| **67** | A(H1N1)pdm09 | A/Virginia/12156/2020 | U.S. Air Force School of Aerospace Medicine | H275Y | Original | EPI1757108 | United States | 2019–2020 |
| **68** | A(H1N1)pdm09 | A/Bangladesh/4005/2019 | Centers for Disease Control and Prevention | H275Y | Original | EPI1608031 | Bangladesh | 2019–2020 |
| **69** | A(H1N1)pdm09 | A/Pennsylvania/115/2020 | Centers for Disease Control and Prevention | H275Y | Original | EPI1877007 | United States | 2019–2020 |
| **70** | A(H1N1)pdm09 | A/Pennsylvania/29/2020 | Centers for Disease Control and Prevention | H275Y | Original | EPI1873431 | United States | 2019–2020 |
| **71** | A(H1N1)pdm09 | A/Texas/136/2020 | Centers for Disease Control and Prevention | H275Y | Original | EPI1799346 | United States | 2019–2020 |
| **72** | A(H1N1)pdm09 | A/Texas/137/2020 | Centers for Disease Control and Prevention | H275Y | Original | EPI1799338 | United States | 2019–2020 |
| **73** | A(H1N1)pdm09 | A/Texas/138/2020 | Centers for Disease Control and Prevention | H275Y | Original | EPI1799330 | United States | 2019–2020 |
| **74** | A(H1N1)pdm09 | A/Texas/164/2020 | Centers for Disease Control and Prevention | H275Y | Original | EPI1873621 | United States | 2019–2020 |
| **75** | A(H1N1)pdm09 | A/Texas/439/2019 | Centers for Disease Control and Prevention | H275Y | Original | EPI1684040 | United States | 2019–2020 |
| **76** | A(H1N1)pdm09 | A/FUKUSHIMA/37/2020 | National Institute of Infectious Diseases (NIID) | H275Y | Original | EPI1854070 | Japan | 2019–2020 |
| **77** | A(H1N1)pdm09 | A/SAGA/262/2019 | National Institute of Infectious Diseases (NIID) | H275Y | Original | EPI1882490 | Japan | 2019–2020 |
| **78** | A(H1N1)pdm09 | A/SAGA/263/2019 | National Institute of Infectious Diseases (NIID) | H275Y | Original | EPI1882500 | Japan | 2019–2020 |
| **79** | A(H1N1)pdm09 | A/SAGA/273/2019 | National Institute of Infectious Diseases (NIID) | H275Y | Original | EPI1882510 | Japan | 2019–2020 |
| **80** | A(H1N1)pdm09 | A/Athens.GR/95/2019 | Hellenic Pasteur Institute | H275Y/H mix | Cell | EPI1358832 | Greece | 2018–2019 |
| **81** | A(H1N1)pdm09 | A/England/22/2019 | Microbiology Reference Services, Colindale, Public Health England | H275Y/H mix | Original | EPI1369932 | United Kingdom | 2018–2019 |
| **82** | A(H1N1)pdm09 | A/England/70/2019 | Microbiology Reference Services, Colindale, Public Health England | H275Y/H mix | Original | EPI1417801 | United Kingdom | 2018–2019 |
| **83** | A(H1N1)pdm09 | A/England/729/2018 | Microbiology Reference Services, Colindale, Public Health England | H275Y/H mix | Original | EPI1356095 | United Kingdom | 2018–2019 |
| **84** | A(H1N1)pdm09 | A/Mexico/1333/2019 | Centers for Disease Control and Prevention | H275Y/H mix | Cell | EPI1547451 | Mexico | 2018–2019 |
| **85** | A(H1N1)pdm09 | A/Stockholm/28/2020 | Public Health Agency Sweden | H275Y/H mix | Original | EPI1709505 | Sweden | 2019–2020 |
| **86** | A(H1N1)pdm09 | A/Stockholm/6/2020 | Public Health Agency Sweden | H275Y/H mix | Original | EPI1682970 | Sweden | 2019–2020 |
| **87** | A(H1N1)pdm09 | A/Massachusetts/50/2019 | Centers for Disease Control and Prevention | H275Y/H mix | Original | EPI1862250 | United States | 2019–2020 |
| **88** | A(H1N1)pdm09 | A/North Carolina/12/2020 | Centers for Disease Control and Prevention | H275Y/H mix | Original | EPI1862569 | United States | 2019–2020 |
| **89** | A(H1N1)pdm09 | A/Netherlands/10095/2019 | National Institute for Public Health and the Environment | D199E/D mix; H275Y/H mix | Original | EPI1377423 | Netherlands | 2018–2019 |
| **90** | A(H1N1)pdm09 | A/Maine/38/2018 | Centers for Disease Control and Prevention | Q136K/Q mix | Cell | EPI1593536 | United States | 2018–2019 |
| **91** | A(H1N1)pdm09 | A/Santa Catarina/331/2019 | Centers for Disease Control and Prevention | Q136R/Q mix | Cell | EPI1628397 | Brazil | 2018–2019 |
| **92** | A(H1N1)pdm09 | A/Tver/12R26/2019^d^ | N.F. Gamaleya Research Center for Epidemiology and Microbiology | R152K | Original | EPI1752545 | Russian Federation | 2018–2019 |
| **93** | A(H1N1)pdm09 | A/Human/Cheboksary/125/2020 | N.F. Gamaleya Research Center for Epidemiology and Microbiology | R152K | Cell | EPI1752521 | Russian Federation | 2019–2020 |
| **94** | A(H1N1)pdm09 | A/England/228/2019 | Crick Worldwide Influenza Centre | D199E | Cell | EPI1575106 | United Kingdom | 2018–2019 |
| **95** | A(H1N1)pdm09 | A/Yunnan-Eshanyizuzizhi/SWL319/2018 | WHO Chinese National Influenza Center | I223M | Cell | EPI1504455 | China | 2018–2019 |
| **96** | A(H1N1)pdm09 | A/Yunnan-Yimen/SWL32/2018 | WHO Chinese National Influenza Center | I223M | Cell | EPI1504458 | China | 2018–2019 |
| **97** | A(H1N1)pdm09 | A/Nepal/19FL3861/2019 | National Institute of Infectious Diseases (NIID) | I223K | Cell | EPI1711343 | Nepal | 2018–2019 |
| **98** | A(H1N1)pdm09 | A/District of Columbia/10770/2020 | U.S. Air Force School of Aerospace Medicine | I223R | Original | EPI1706206 | United States | 2019–2020 |
| **99** | A(H1N1)pdm09 | A/Saint-Petersburg/RII-540S/2019 | Research Institute of Influenza, Ministry of Healthcare of the Russian Federation | I223T | Original | EPI1653166 | Russian Federation | 2019–2020 |
| **100** | A(H1N1)pdm09 | A/Tennessee/88/2019 | Centers for Disease Control and Prevention | I223T | Original | EPI1860474 | United States | 2019–2020 |
| **101** | A(H1N1)pdm09 | A/Khabarovsk/63/2019 | State Research Centre for Virology and Biotechnology VECTOR | I223T | Cell | EPI1423072 | Russian Federation | 2018–2019 |
| **102** | A(H1N1)pdm09 | A/Hunan-Hecheng/SWL1350/2019 | WHO Chinese National Influenza Center | I223T; S247N | Cell | EPI1648843 | China | 2018–2019 |
| **103** | A(H1N1)pdm09 | A/Xinjiang-Aletai/SWL132/2019 | WHO Chinese National Influenza Center | S247G | Cell | EPI1504392 | China | 2018–2019 |
| **104** | A(H1N1)pdm09 | A/Oklahoma/10319/2019 | U.S. Air Force School of Aerospace Medicine | S247G | Original | EPI1667910 | United States | 2019–2020 |
| **105** | A(H1N1)pdm09 | A/South Korea/11256/2020 | U.S. Air Force School of Aerospace Medicine | S247G | Original | EPI1709875 | Republic of Korea | 2019–2020 |
| **106** | A(H1N1)pdm09 | A/Pennsylvania/276/2020 | Centers for Disease Control and Prevention | N295S | Original | EPI1869948 | United States | 2019–2020 |
|  |  |  |  |  |  |  |  |  |
| **1** | A(H3N2) | A/Bolzano/12/2019 | Istituto Superiore di Sanità | E119V | Other | EPI1640161 | Italy | 2018–2019 |
| **2** | A(H3N2) | A/Zaragoza/493/2018 | Crick Worldwide Influenza Centre | E119V | Original | EPI1315710 | Spain | 2018–2019 |
| **3** | A(H3N2) | A/England/195040727/2019 | Microbiology Reference Services, Colindale, Public Health England | E119V | Original | EPI1679256 | United Kingdom | 2019–2020 |
| **4** | A(H3N2) | A/Zaragoza/1528894/2018 | Crick Worldwide Influenza Centre | E119V/E mix | Original | EPI1315678 | Spain | 2018–2019 |
| **5** | A(H3N2) | A/Zaragoza/492/2018 | Crick Worldwide Influenza Centre | E119V/E mix | Original | EPI1315704 | Spain | 2018–2019 |
| **6** | A(H3N2) | A/Zaragoza/489/2018 | Crick Worldwide Influenza Centre | E119V/E mix | Original | EPI1315680 | Spain | 2018–2019 |
| **7** | A(H3N2) | A/Singapore/TT0889/2019 | Ministry of Health, Singapore | E119G/E mix | Original | EPI1646383 | Singapore | 2019–2020 |
| **8** | A(H3N2) | A/Netherlands/10250/2019 | National Institute for Public Health and the Environment | K249E | Original | EPI1423240 | Netherlands | 2018–2019 |
| **9** | A(H3N2) | A/Singapore/GP2153/2019 | Ministry of Health, Singapore | Q391K | Original | EPI1646173 | Singapore | 2019–2020 |
| **10** | A(H3N2) | A/Finland/140/2019 | National Institute for Health and Welfare | R292K | Other | EPI1745734 | Finland | 2019–2020 |
|  |  |  |  |  |  |  |  |  |
| **1** | B/Vic | B/Virginia/11839/2020 | U.S. Air Force School of Aerospace Medicine | Y142H | Original | EPI1738614 | United States | 2019–2020 |
| **2** | B/Vic | B/Romania/798/2020 | National Institute of Infectious Diseases | G145E | Original | EPI1584222 | Romania | 2019–2020 |
| **3** | B/Vic | B/Pennsylvania/77/2020 | Centers for Disease Control and Prevention | D197N | Cell | EPI1867006 | United States | 2018–2019 |
| **4** | B/Vic | B/CastillaLaMancha/1090/2020 | Crick Worldwide Influenza Centre | D197N | Original | EPI1800694 | Spain | 2018–2019 |
| **5** | B/Vic | B/District of Columbia/08/2020 | Centers for Disease Control and Prevention | D197N | Original | EPI1840575 | United States | 2018–2019 |
| **6** | B/Vic | B/Bangladesh/1730/2019 | Centers for Disease Control and Prevention | D197N | Original | EPI1618569 | Bangladesh | 2019–2020 |
| **7** | B/Vic | B/Alberta/RV5264/2020 | Public Health Agency of Canada (PHAC) | I221T | Cell | EPI1745774 | Canada | 2019–2020 |
| **8** | B/Vic | B/Idaho/12058/2020 | U.S. Air Force School of Aerospace Medicine | I221T | Original | EPI1745205 | United States | 2019–2020 |
| **9** | B/Vic | B/Argentina/2839/2019 | Centers for Disease Control and Prevention | K360E | Original | EPI1650756 | Argentina | 2019–2020 |
| **10** | B/Vic | B/Argentina/2874/2019 | Centers for Disease Control and Prevention | K360E | Original | EPI1650705 | Argentina | 2019–2020 |
| **11** | B/Vic | B/Parana/115/2019 | Instituto Oswaldo Cruz FIOCRUZ—Laboratory of Respiratory Viruses and Measles (LVRS) | K360E | Original | EPI1327522 | Brazil | 2018–2019 |
| **12** | B/Vic | B/Argentina/61/2020 | Centers for Disease Control and Prevention | K360E | Original | EPI1392826 | Argentina | 2018–2019 |
| **13** | B/Vic | B/Argentina/100/2020 | Centers for Disease Control and Prevention | K360E | Original | EPI1752168 | Argentina | 2018–2019 |
|  |  |  |  |  |  |  |  |  |
| **1** | B/Yam | B/Concepcion/81421/2018 | Instituto de Salud Pública de Chile | G145E | Original | EPI1731521 | Chile | 2018–2019 |
| **2** | B/Yam | B/Texas/03/2019 | Centers for Disease Control and Prevention | S246P | Cell | EPI1368656 | United States | 2018–2019 |

GISAID: Global Initiative on Sharing All Influenza Data; HRI: highly reduced inhibition; RI: reduced inhibition.

^a^ A total of 15079 and 10428 NA sequences for 2018-2019 and 2019-2020, respectively, were obtained from GISAID and analyzed for NA amino acid substitutions conferring RI/HRI phenotypes. To ensure accuracy, the sequences were curated to remove duplicate sequences for individual viruses and preference was given to the sequence from the original clinical specimen, if available.

^b^ NA amino acid numbering is A-subtype or B-type specific. NA amino acid substitutions associated with RI/HRI, as listed in the summary table provided by the AVWG on the WHO website ([https://cdn.who.int/media/docs/default-source/influenza/nai-reduced-susceptibility-marker-table-who_table-1.pdf](https://cdn.who.int/media/docs/default-source/influenza/nai-reduced-susceptibility-marker-table-who_table-1.pdf?sfvrsn=7ff5a545_1&download=true)), are shown.

^c^ Passage as shown in the sequence databases.

^d^ Virus name in GISAID entered as A/Human/Tver/12R26/2019.

**Table S6.** Influenza types A and B viruses carrying PA substitutions potentially associated with reduced susceptibility to baloxavir (n=90).^a^

| **#** | **Type/subtype or lineage** | **Strain designation** | **Submitting laboratory** | **EC_50_ fold-change as compared to reference**  **median EC_50_^b^** | **PA substitution in virus isolate^c^** | **PA substitution in original specimen^c^** | **Patient setting** | **Antiviral treatment** | **Immuno-compromised** | **Country of specimen collection** | **Date of collection (y/m/d)** | **Age (years)** | **GISAID PA accession #** |
| --- | --- | --- | --- | --- | --- | --- | --- | --- | --- | --- | --- | --- | --- |
| **2018-2019 period** | | | | | | | | | | | | | |
| 1 | A(H1N1)pdm09 | A/Kanagawa/AC1830/2019 | Tokyo | 32.2 | I38T | I38T/I mix | Community | Yes, baloxavir | No | Japan | 2019/01/15 | 14 | EPI1422882 |
| 2 | A(H1N1)pdm09 | A/Kanagawa/IC1890/2019 | Tokyo | 11.4 | I38T | I38T/I mix | Community | Yes, baloxavir | No | Japan | 2019/01/28 | 9 | EPI1384002 |
| 3 | A(H1N1)pdm09 | A/South Korea/8354/2019 | U.S. Air Force School of Aerospace Medicine | n/t^d^ | Not available | I38T | Unknown | Unknown | Unknown | Republic of Korea | 2019/01/04 | 41 | EPI1519150 |
| 4 | A(H1N1)pdm09 | A/Kanagawa/88/2018 | Tokyo | 12.4 | I38T/F mix | I38T/F/I mix | Community | Yes, baloxavir | No | Japan | 2018/11/20 | 8 | EPI1383947 |
| 5 | A(H1N1)pdm09 | A/Wakayama/21/2019 | Tokyo | 2.5 | I38F/I mix | I38F/I mix | Unknown | Unknown | Unknown | Japan | 2019/02/04 | 9 | EPI1393833 |
| 6 | A(H1N1)pdm09 | A/Yokohama/122/2019 | Tokyo | 36.9 | I38S | I38S | Community | Yes, baloxavir | No | Japan | 2019/02/13 | 6 | EPI1716575 |
| 7 | A(H1N1)pdm09 | A/Yokohama/98/2019 | Tokyo | 49.5 | I38S | I38S | Community | Yes, baloxavir | No | Japan | 2019/02/08 | 6 | EPI1444656 |
| 8 | A(H1N1)pdm09 | A/Ibaraki/37/2018 | Tokyo | 3.6 | I38V | I38V | Community | Yes, laninamivir | No | Japan | 2018/12/15 | 7 | EPI1610320 |
| 9 | A(H1N1)pdm09 | A/Ibaraki/38/2018 | Tokyo | 3.7 | I38V | I38V | Community | Yes, laninamivir | No | Japan | 2019/01/09 | 6 | EPI1610323 |
| 10 | A(H1N1)pdm09 | A/Ibaraki/42/2018 | Tokyo | 3 | I38V | I38V | Community | Yes, laninamivir | No | Japan | 2019/01/24 | 38 | EPI1610326 |
| 11 | A(H1N1)pdm09 | A/Washington/191/2019 | Atlanta | n/t | Not available | I38V | Unknown | Unknown | Unknown | United States | 2019/02/08 | 38 | EPI1558625 |
| 12 | A(H1N1)pdm09 | A/California/98/2018 | Atlanta | n/t | Not available | I38V | Unknown | Unknown | Unknown | United States | 2018/12/01 | 57 | EPI1337856 |
| 13 | A(H1N1)pdm09 | A/California/117/2018 | Atlanta | n/t | Not available | I38V | Unknown | Unknown | Unknown | United States | 2018/12/21 | 34 | EPI1349328 |
| 14 | A(H1N1)pdm09 | A/California/NHRC-OID FDX100117/2018 | Naval Health Research Center | n/t | I38V | Not available | Unknown | Unknown | Unknown | United States | 2018/10/03 | unknown | EPI1523709 |
| 15 | A(H1N1)pdm09 | A/California/NHRC-OID_FDX100165/2018 | Naval Health Research Center | n/t | I38V | Not available | Unknown | Unknown | Unknown | United States | 2018/11/20 | unknown | EPI1523717 |
| 16 | A(H1N1)pdm09 | A/California/NHRC-OID_FDX120099/2018 | Naval Health Research Center | n/t | I38V | Not available | Unknown | Unknown | Unknown | United States | 2018/12/12 | unknown | EPI1501663 |
| 17 | A(H1N1)pdm09 | A/Washington/9308/2019 | United States Air Force | n/t | I38V | Not available | Unknown | Unknown | Unknown | United States | 2019/02/05 | 61 | EPI1770705 |
| 18 | A(H1N1)pdm09 | A/Arizona/35/2018 | Atlanta | 6.9 | E23G | E23G | Unknown | Unknown | Unknown | United States | 2019/01/28 | 8 | EPI1254748 |
| 19 | A(H1N1)pdm09 | A/West Virginia/02/2019 | Atlanta | 4.7 [4.1] | K34R | K34R | Unknown | Unknown | Unknown | United States | 2019/01/02 | 8 | EPI1363590 |
| 20 | A(H1N1)pdm09 | A/Saitama/149/2018 | Tokyo | 1.6 | K34R | Not available | Unknown | Unknown | Unknown | Japan | 2018/12/07 | 11 | EPI1381295 |
| 21 | A(H1N1)pdm09 | A/MASSACHUSETTS/06/2019 | Atlanta | 2.90 [3.7] | E199G | E199G | Unknown | Unknown | Unknown | United States | 2019/01/24 | 53 | EPI1574297 |
|  |  |  |  |  |  |  |  |  |  |  |  |  |  |
| 22 | A(H3N2) | A/Kanagawa/IC18144/2019 | Tokyo | n/t | Not available | I38T | Unknown | Unknown | Unknown | Japan | 2019/02/09 | 10 | EPI1389907 |
| 23 | A(H3N2) | A/Hiroshima-C/30/2019 | Tokyo | 89.2 | I38T | I38T | Community | Yes, baloxavir | No | Japan | 2019/04/17 | 2 | EPI1436220 |
| 24 | A(H3N2) | A/Kanagawa/AC1878/2019 | Tokyo | 88.1 | I38T | I38T | Community | Yes, baloxavir | No | Japan | 2019/03/14 | 6 | EPI1444720 |
| 25 | A(H3N2) | A/Kanagawa/IC18141/2019 | Tokyo | 129 | I38T | I38T | Community | No | No | Japan | 2019/02/12 | 0 | EPI1383986 |
| 26 | A(H3N2) | A/Kanagawa/IC1894/2019 | Tokyo | 85.8 | I38T | I38T | Community | Yes, baloxavir | No | Japan | 2019/01/31 | 13 | EPI1384010 |
| 27 | A(H3N2) | A/Kobe/18578/2019 | Tokyo | 171 | I38T | I38T | Community | Yes, baloxavir | No | Japan | 2019/03/05 | 4 | EPI1656079 |
| 28 | A(H3N2) | A/Mie/41/2018 | Tokyo | 384 | I38T | I38T | Community | No | No | Japan | 2019/02/21 | 12 | EPI1389867 |
| 29 | A(H3N2) | A/Yokohama/133/2018 | Tokyo | 124 | I38T | I38T | Community | Yes, baloxavir | No | Japan | 2018/12/14 | 6 | EPI1333662 |
| 30 | A(H3N2) | A/Yokohama/56/2019 | Tokyo | 234 | I38T | I38T | Community | Yes, baloxavir | No | Japan | 2019/01/15 | 1 | EPI1366249 |
| 31 | A(H3N2) | A/Yokohama/88/2019 | Tokyo | 120 | I38T | I38T | Hospital | Yes, oseltamivir | No | Japan | 2019/02/15 | 5 | EPI1370455 |
| 32 | A(H3N2) | A/Kanagawa/IC1807/2018 | Tokyo | 70.1 | I38T | I38T/I mix | Community | Yes, baloxavir | No | Japan | 2018/12/25 | 14 | EPI1366189 |
| 33 | A(H3N2) | A/Kanagawa/IC18102/2019 | Tokyo | 64.3 | I38T | I38T/I mix | Community | Yes, baloxavir | No | Japan | 2019/01/31 | 11 | EPI1384018 |
| 34 | A(H3N2) | A/Kanagawa/IC18160/2019 | Tokyo | 140 | I38T | I38T/I mix | Community | Yes, baloxavir | No | Japan | 2019/02/25 | 10 | EPI1381313 |
| 35 | A(H3N2) | A/Kanagawa/IC1861/2019 | Tokyo | 122 | I38T | I38T/I mix | Community | Yes, baloxavir | Unknown | Japan | 2019/01/24 | 14 | EPI1413447 |
| 36 | A(H3N2) | A/Kanagawa/IC1869/2019 | Tokyo | 263 | I38T | I38T/I mix | Community | Yes, baloxavir | No | Japan | 2019/01/28 | 7 | EPI1383955 |
| 37 | A(H3N2) | A/Wakayama/18/2019 | Tokyo | 79.2 | I38T | I38T/I mix | Community | Yes, baloxavir | No | Japan | 2019/02/27 | 14 | EPI1381317 |
| 38 | A(H3N2) | A/Wakayama/23/2019 | Tokyo | 131 | I38T | I38T/I mix | Community | Yes, baloxavir | No | Japan | 2019/02/27 | 3 | EPI1381325 |
| 39 | A(H3N2) | A/Yokohama/135/2018 | Tokyo | 78.7 | I38T | I38T/I mix | Community | Yes, baloxavir | No | Japan | 2018/12/14 | 7 | EPI1329067 |
| 40 | A(H3N2) | A/Yokohama/87/2019 | Tokyo | 86.3 | I38T | I38T/I mix | Hospital | Yes, baloxavir | No | Japan | 2019/02/15 | 6 | EPI1370439 |
| 41 | A(H3N2) | A/Kanagawa/IC18143/2019 | Tokyo | 102 | I38T | I38T/K/I mix | Community | Yes, baloxavir | No | Japan | 2019/02/12 | 7 | EPI1383994 |
| 42 | A(H3N2) | A/Tokyo/18500/2019 | Tokyo | 95.1 | I38T | Not available | Community | No | No | Japan | 2019/01/30 | 86 | EPI1593915 |
| 43 | A(H3N2) | A/Kanagawa/AC1829/2019 | Tokyo | 18 | I38T/I mix | I38T/I mix | Community | Yes, baloxavir | No | Japan | 2019/01/17 | 4 | EPI1353544 |
| 44 | A(H3N2) | A/Kanagawa/IC18103/2019 | Tokyo | 5.6 | I38T/I mix | I38T/I mix | Community | Yes, baloxavir | No | Japan | 2019/01/31 | 8 | EPI1447295 |
| 45 | A(H3N2) | A/Kanagawa/IC1827/2019 | Tokyo | 72.1 | I38T/I mix | I38T/I mix | Community | Yes, baloxavir | No | Japan | 2019/01/15 | 5 | EPI1353552 |
| 46 | A(H3N2) | A/Wakayama/19/2019 | Tokyo | 112 | I38T/I mix | I38T/I mix | Community | Yes, baloxavir | No | Japan | 2019/02/27 | 6 | EPI1384052 |
| 47 | A(H3N2) | A/Wakayama/22/2019 | Tokyo | 31 | I38T/I mix | I38T/I mix | Community | Yes, baloxavir | No | Japan | 2019/02/27 | 2 | EPI1381321 |
| 48 | A(H3N2) | A/Yokohama/61/2019 | Tokyo | 250 | I38T/I mix | I38T/I mix | Community | Yes, baloxavir | No | Japan | 2019/02/07 | 4 | EPI1366290 |
| 49 | A(H3N2) | A/Yokohama/99/2019 | Tokyo | 63.3 | I38T/I mix | I38T/I mix | Community | Yes, baloxavir | No | Japan | 2019/02/22 | 6 | EPI1937521 |
| 50 | A(H3N2) | A/Kanagawa/IC1817/2019 | Tokyo | 25.6 | I38T/M/I mix | I38T/M/I mix | Community | Yes, baloxavir | No | Japan | 2019/01/10 | 9 | EPI1366227 |
| 51 | A(H3N2) | A/Basel/5570/2018 | University Hospital Basel | n/t | I38L/V/I mix | Not available | Unknown | Unknown | Unknown | Switzerland | 2018/11/16 | unknown | EPI1331967 |
| 52 | A(H3N2) | A/Washington/636/2019 | Atlanta | n/t | Not available | I38M | Unknown | Unknown | Unknown | United States | 2019/04/10 | 25 | EPI1562216 |
| 53 | A(H3N2) | A/Kanagawa/IC1870/2019 | Tokyo | 23.7 | I38M | I38M | Community | Yes, baloxavir | No | Japan | 2019/01/28 | 11 | EPI1383963 |
| 54 | A(H3N2) | A/Massachusetts/04/2019 | Atlanta | 1.04 [1.2] | I38M/I mix | I38 | Unknown | Unknown | Unknown | United States | 2019/01/15 | 7 | EPI1397514 |
| 55 | A(H3N2) | A/Kanagawa/AC1817/2018 | Tokyo | 91.8 | I38M/I mix | I38M/I mix | Community | Yes, baloxavir | No | Japan | 2018/12/27 | 8 | EPI1353496 |
| 56 | A(H3N2) | A/Belgium/S0476/2019 | SCIENSANO | n/t | Not available | I38V | Unknown | Unknown | Unknown | Belgium | 2019/01/29 | 67 | EPI1446404 |
| 57 | A(H3N2) | A/Wisconsin/444/2019 | Atlanta | n/t | I38V/I mix | Not available | Unknown | Unknown | Unknown | United States | 2019/03/25 | 13 | EPI1560406 |
| 58 | A(H3N2) | A/St. Vincent And Grenadines/3612/2019 | Atlanta | 0.9  [0.80] | I38V/I mix | Not available | Unknown | Unknown | Unknown | Saint Vincent and the Grenadines | 2019/04/15 | 3 | EPI1591545 |
| 59 | A(H3N2) | A/Congo/732/2018 | Atlanta | 4.1 [3.7] | K34R | K34R | Unknown | Unknown | Unknown | Democratic Republic of Congo | 2018/12/27 | 6 | EPI1386869 |
| 60 | A(H3N2) | A/Italy/8602/2019 | U.S. Air Force School of Aerospace Medicine | n/t | Not available | L28P | Unknown | Unknown | Unknown | Italy | 2019/01/25 | 41 | EPI1499916 |
| 61 | A(H3N2) | A/New Jersey/7720/2019 | U.S. Air Force School of Aerospace Medicine | n/t | Not available | L28P | Unknown | Unknown | Unknown | United States | 2019/01/02 | 23 | EPI1524786 |
| 62 | A(H3N2) | A/New Jersey/7721/2018 | U.S. Air Force School of Aerospace Medicine | n/t | Not available | L28P | Unknown | Unknown | Unknown | United States | 2018/12/30 | 18 | EPI1524794 |
| 63 | A(H3N2) | A/New Jersey/7722/2018 | U.S. Air Force School of Aerospace Medicine | n/t | Not available | L28P | Unknown | Unknown | Unknown | United States | 2018/12/30 | 21 | EPI1524802 |
| 64 | A(H3N2) | A/New Jersey/7723/2018 | U.S. Air Force School of Aerospace Medicine | n/t | Not available | L28P | Unknown | Unknown | Unknown | United States | 2018/12/28 | 19 | EPI1524571 |
| 65 | A(H3N2) | A/New Jersey/7729/2019 | U.S. Air Force School of Aerospace Medicine | n/t | Not available | L28P | Unknown | Unknown | Unknown | United States | 2019/01/03 | 35 | EPI1524850 |
| 66 | A(H3N2) | A/New York/7735/2018 | U.S. Air Force School of Aerospace Medicine | n/t | Not available | L28P | Unknown | Unknown | Unknown | United States | 2018/12/13 | 21 | EPI1524882 |
| 67 | A(H3N2) | A/New York/7736/2018 | U.S. Air Force School of Aerospace Medicine | n/t | Not available | L28P | Unknown | Unknown | Unknown | United States | 2018/12/14 | 32 | EPI1524890 |
| 68 | A(H3N2) | A/New York/7739/2018 | U.S. Air Force School of Aerospace Medicine | n/t | Not available | L28P | Unknown | Unknown | Unknown | United States | 2018/12/19 | 19 | EPI1524906 |
| 69 | A(H3N2) | A/Ohio/7751/2018 | U.S. Air Force School of Aerospace Medicine | n/t | Not available | L28P | Unknown | Unknown | Unknown | United States | 2018/12/21 | 1 | EPI1524970 |
| 70 | A(H3N2) | A/South Australia/85/2018 | Victorian Infectious Diseases Reference Laboratory | n/t | Not available | L28P | Unknown | Unknown | Unknown | Australia | 2018/09/13 | 15 | EPI1369997 |
| 71 | A(H3N2) | A/Washington/9447/2019 | U.S. Air Force School of Aerospace Medicine | n/t | Not available | L28P | Unknown | Unknown | Unknown | United States | 2019/02/24 | 74 | EPI1577267 |
| 72 | A(H3N2) | A/St Clears/8157/2019 | Public Health Wales Microbiology Cardiff | n/t | Not available | E23K+K34Q | Unknown | Unknown | Unknown | United Kingdom | 2019/04/17 | 92 | EPI1444586 |
| **2019-2020 period** | | | | | | | | | | | | | |
| 1 | A(H1N1)pdm09 | A/Kansas/11881/2020 | U.S. Air Force School of Aerospace Medicine | n/t | Not available | I38V | Unknown | Unknown | Unknown | United States | 2020/02/11 | 28 | EPI1735675 |
| 2 | A(H1N1)pdm09 | A/Kanagawa/AC1920/2019 | Tokyo | 7.4 | E23K | E23K | Community | No | No | Japan | 2019/12/01 | 10 | EPI1679821 |
|  |  |  |  |  |  |  |  |  |  |  |  |  |  |
| 3 | A(H3N2) | A/Aichi/118/2019 | Tokyo | 614 | I38T | I38T | Community | Yes, baloxavir | No | Japan | 2019/07/09 | 38 | EPI1593918 |
| 4 | A(H3N2) | A/Michigan/390/2019 | Atlanta | 4.1 [7.9] | I38L | I38L | Unknown | Unknown | Unknown | United States | 2019/08/07 | 72 | EPI1548538 |
| 5 | A(H3N2) | A/Berlin/9/2020 | Robert Koch Institute | n/t | Not available | I38M | Unknown | Unknown | Unknown | Germany | 2020/01/18 | 66 | EPI1690636 |
| 6 | A(H3N2) | A/Florida/11504/2020 | U.S. Air Force School of Aerospace Medicine | n/t | Not available | L28P | Unknown | Unknown | Unknown | United States | 2020/02/03 | 4 | EPI1729174 |
| 7 | A(H3N2) | A/Toamasina/00356/2020 | Crick Worldwide Influenza Centre | n/t | L28P | Not available | Unknown | Unknown | Unknown | Madagascar | 2020/01/29 | unknown | EPI1846359 |
| 8 | A(H3N2) | A/California/142/2019 | Atlanta | 0.48 [0.40] | L28P | L28P | Unknown | Unknown | Unknown | United States | 2019/05/22 | 11 | EPI1482853 |
| 9 | A(H3N2) | A/Peru/113154619/2019 | Atlanta | 1.3 [1.0] | L28P | Not available | Unknown | Unknown | Unknown | Peru | 2019/11/15 | 0 | EPI1702598 |
| 10 | A(H3N2) | A/Talca/63220/2019 | Atlanta | n/t | E199G | Not available | Unknown | Unknown | Unknown | Chile | 2019/07/04 | 7 | EPI1587653 |
|  |  |  |  |  |  |  |  |  |  |  |  |  |  |
| 11 | B/Victoria | B/Lebanon/12/2020 | Atlanta | 0.88 [1.5] | I38V | I38V | Unknown | Unknown | Unknown | Lebanon | 2020/01/29 | 5 | EPI1806626 |
| 12 | B/Victoria | B/Nevada/9939/2019 | U.S. Air Force School of Aerospace Medicine | n/t | Not available | I38V | Unknown | Unknown | Unknown | United States | 2019/11/08 | 11 | EPI1654626 |
| 13 | B/Victoria | B/Saint-Petersburg/RII-2408S/2020 | Research Institute of Influenza, Ministry of Healthcare of the Russian Federation | n/t | Not available | I38V | Unknown | Unknown | Unknown | Russian Federation | 2020/02/17 | 22 | EPI1721015 |
| 14 | B/Victoria | B/Fukushima/1/2020 | Tokyo | 0.6 | M34I | Not available | Unknown | Unknown | Unknown | Japan | 2020/01/27 | 5 | EPI1806911 |
| 15 | B/Victoria | B/Georgia/10955/2020 | U.S. Air Force School of Aerospace Medicine | n/t | Not available | M34I | Unknown | Unknown | Unknown | United States | 2020/01/07 | 13 | EPI1707529 |
| 16 | B/Victoria | B/Lebanon/120/2020 | Atlanta | n/t | Not available | M34V | Unknown | Unknown | Unknown | Lebanon | 2020/01/24 | 40 | EPI1726010 |
| 17 | B/Victoria | B/New Jersey/12075/2020 | U.S. Air Force School of Aerospace Medicine | n/t | Not available | M34I | Unknown | Unknown | Unknown | United States | 2020/03/02 | 36 | EPI1745306 |
| 18 | B/Victoria | B/Stockholm/11/2020 | Public Health Agency of Sweden / Department of Microbiology | n/t | Not available | M34I | Unknown | Unknown | Unknown | Sweden | 2020/03/02 | 5 | EPI1722747 |

^a^ Viruses were analyzed for susceptibility to baloxavir, using sequence-based, phenotypic, or both methods.

^b^ Fold-change in susceptibility to baloxavir compared to PA sequence-matched control virus is shown in [brackets] for viruses tested at Atlanta WHO CC.

^c^ PA amino acid substitutions potentially associated with reduced susceptibility to baloxavir, as listed in the summary table provided by the AVWG on the WHO website (<https://cdn.who.int/media/docs/default-source/influenza/summary-of-polymerase-acidic-(pa)-protein-amino-acid-substitutions-analysed-for-their-effects-on-baloxavir-susceptibility.pdf>) are shown. Substitutions at PA residue 34 are included.

^d^ n/t: not tested.

**Table S7**. Zoonotic influenza A viruses (n = 14) carrying PA substitutions associated with reduced susceptibility to baloxavir.

| Influenza A virus subtype (clade) | n | Geographical area of virus isolation (n) | Amino acid substitutions associated with reduced antiviral susceptibility | | GISAID accession number for identified substitutions  (PA gene) |
| --- | --- | --- | --- | --- | --- |
|  |  |  | NA substitution | PA  substitution |  |
| *Swine influenza* | | | | | |
| \| H1N1v (pdm09; 6B.1A) \| \| --- \| | 1 | USA (1) | None^a^ | None | N/A^b^ |
| \|  \| \| --- \|   H1N2v (delta 2, 1B.2) | 10 | Brazil (1), USA (9) | None | None | N/A |
| H3N2v | 1 | USA (1) | None | None | N/A |
| *Avian influenza* | | | | | |
| H5N1 (2.3.2.1a) (HPAI^c^) | 1 | Nepal (1) | None | None | N/A |
| H5N6 (2.3.4.4) (HPAI) | 4 | China (4) | None | A37S (2)  I38V+E199G (1) | EPI1352801, EPI1352809, EPI1352817 |
| H7N9 (HPAI) | 1 | China (1) | None | A37S (1) | EPI1431588 |
| H9N2 | 14 | China (10), India (1),  HK SAR (1), Oman (1), Senegal (1) | None | A37S (10) | EPI1384080, EPI1384088, EPI1676301, EPI1694143,  EPI1738878, EPI1804955, EPI1804949, EPI1804941,  EPI1815741, EPI1815741 |

^a^ None: no substitutions previously reported to be associated with reduced susceptibility to NAIs or PAI were identified.

^b^ N/A: not applicable.

^c^ Highly Pathogenic Avian Influenza virus.
